# Developmental constraint shaped genome evolution and erythrocyte loss in Antarctic fishes following paleoclimate change

**DOI:** 10.1371/journal.pgen.1009173

**Published:** 2020-10-27

**Authors:** Jacob M. Daane, Juliette Auvinet, Alicia Stoebenau, Donald Yergeau, Matthew P. Harris, H. William Detrich

**Affiliations:** 1 Department of Marine and Environmental Sciences, Northeastern University Marine Science Center, Nahant, MA, United States of America; 2 Orthopaedic Research Laboratories, Department of Orthopaedic Surgery, Boston Children's Hospital, Boston, MA, United States of America; 3 Department of Genetics, Harvard Medical School, Boston, MA, United States of America; 4 Department of Biology, Northeastern University, Boston, MA, United States of America; University of Pennsylvania School of Medicine, UNITED STATES

## Abstract

In the frigid, oxygen-rich Southern Ocean (SO), Antarctic icefishes (Channichthyidae; Notothenioidei) evolved the ability to survive without producing erythrocytes and hemoglobin, the oxygen-transport system of virtually all vertebrates. Here, we integrate paleoclimate records with an extensive phylogenomic dataset of notothenioid fishes to understand the evolution of trait loss associated with climate change. In contrast to buoyancy adaptations in this clade, we find relaxed selection on the genetic regions controlling erythropoiesis evolved only after sustained cooling in the SO. This pattern is seen not only within icefishes but also occurred independently in other high-latitude notothenioids. We show that one species of the red-blooded dragonfish clade evolved a spherocytic anemia that phenocopies human patients with this disease via orthologous mutations. The genomic imprint of SO climate change is biased toward erythrocyte-associated conserved noncoding elements (CNEs) rather than to coding regions, which are largely preserved through pleiotropy. The drift in CNEs is specifically enriched near genes that are preferentially expressed late in erythropoiesis. Furthermore, we find that the hematopoietic marrow of icefish species retained proerythroblasts, which indicates that early erythroid development remains intact. Our results provide a framework for understanding the interactions between development and the genome in shaping the response of species to climate change.

## Author summary

Our climate is rapidly changing. To better understand how species can adapt to major climate disturbance, we looked back into the past at a group of fishes that have encountered dramatic climate upheavals and thrived: Antarctic notothenioid fishes. In particular, we focus on the icefishes, which lost the ability to produce red blood cells in the frigid environment of the Southern Ocean. By integrating past climate records with a large genetic dataset of Antarctic fishes, we show that the loss of red blood cells occurred only after sustained cooling of the Southern Ocean. As cooling continued into the modern era, we discover that even some of the “red-blooded” relatives of the icefishes show early genetic and morphological signs of erythrocyte loss. This cooling event left a non-random imprint on the genome of icefishes. With few exceptions, the genetic toolkit underlying red cell development has remained intact in icefishes because many “erythroid” genes perform important functions in other tissues. Rather, mutations have accumulated in gene regulatory regions near genes that control terminal erythroid maturation, such that icefishes continue to produce red cell progenitors but not mature erythrocytes. These results show that the genetic constraints regulating embryonic development shaped the evolutionary response of this fish group to climate change.

## Introduction

The cooling of the Southern Ocean (SO) beginning 35 million years ago (Ma) had a profound impact on the evolution of Antarctic fishes [1]. The stable, freezing temperatures, strong currents, and frequent storms created an environment in which dissolved oxygen was abundant and well mixed throughout the water column. In this unique environment, a single clade of Antarctic fishes, the icefishes (Notothenioidei: Cryonotothenioidea: Channichthyidae), lost the capacity to produce erythrocytes and the oxygen-transport protein hemoglobin (Hb)–and yet they thrive in the SO. The connection between paleoclimatic change in the SO and the origins of novel traits in notothenioid fishes provides a natural experiment for understanding the developmental and genetic mechanisms that shape phenotypic responses to environmental change.

Whereas the loss of erythrocytes among vertebrates is unique to icefishes, many closely related, but red-blooded, cryonotothenioid species cohabit the SO. Although having erythrocytes, these red-blooded species show a phylogenetic trend toward reduced hematocrit and/or mean corpuscular hemoglobin concentration, decreased hemoglobin multiplicity, and lowered hemoglobin affinity for O_2_ as one proceeds from basal clades to the crown group Channichthyidae [1–4]. Intriguingly, several red-blooded Antarctic notothenioids survive experimentally induced anemia. Treatment of the bullhead notothen, *Notothenia coriiceps*, with the hemolytic agent phenylhydrazine reduces the percentage of erythrocytes in blood from 35% to 4% without lethality [5]. Similarly, the notothen, *Trematomus bernacchii*, survives conversion of its hemoglobin to the inactive carbonmonoxy state (95% CO-Hb)[6]; in contrast, CO-Hb exceeding 40% is lethal in humans [7]. Thus, erythrocytes and hemoglobin appear to be dispensable in red-blooded notothenioid lineages, which suggests an inherent resiliency within cryonotothenioids to accommodate extreme anemia.

Recent studies of the erythroid system in notothenioids have focused on the evolution of specific candidate genes, most notably the *alpha*- and *beta-globin* genes of the teleost *globin* clusters. These genes are almost completely deleted from the genomes of most icefishes [8–10], and globin regulatory elements are progressively deleted in the ancestral lineages leading to the icefishes [11]. Myoglobin expression is also absent from the hearts of 6 out of 16 icefish species, although mutated myoglobin genes remain in their genomes [12–15]. Furthermore, several genes encoding hemoglobin scavenging proteins, such as *haptoglobin*, have accumulated deleterious mutations and are expressed at reduced levels by icefishes [16]. Early work by Hureau *et al* [17] and by Barber *et al* [18] revealed that icefishes possess small numbers of senescent, "erythrocyte-like" cells that are devoid of hemoglobin. These results suggest that despite the loss of hemoglobin, the block to erythropoiesis in icefishes might be constrained, or incomplete, leading to a minimally functional erythroid genetic program.

Together, these results provide insights into the evolution of the notothenioid hematological system, but a comprehensive assessment of changes to the erythroid developmental and genetic program is lacking. Part of this limitation has been the lack of the genome-wide data across cryonotothenioids necessary to establish a timeline of genomic changes supporting and potentially driving phenotypic adaptations. Recently, we published a dataset of ~250,000 loci representing protein-coding exons and conserved non-coding elements (CNEs) from 44 notothenioid species, including 10 icefishes and 6 dragonfishes (**S1 Fig**) [8]. The power of this phylogeny-wide genomic dataset lies in the ability to reconstruct the genetic steps that preceded, initiated, and follow trait evolution.

In this report, we systematically investigate the evolutionary genetic response of notothenioids to global paleoenvironmental change and explore the preconditions and consequences of erythrocyte loss on icefish genomes. Using these datasets, we discover pronounced shifts to the evolutionary rate of erythrocyte-associated CNEs following global cooling after the mid-Miocene climate transition, and we identify patterns of drift within these regions in extant high-latitude cryonotothenioids. We further demonstrate the retention of the majority of the erythroid protein coding toolkit and the presence of erythroid progenitors in icefish hematopoietic tissues, which together indicate that developmental mechanisms have been maintained through pleiotropy following trait loss.

## Results

Previous studies have focused on the end-point—the status of extant icefishes—but have lacked the genome-wide data from a sufficiently large sample of species to support high-resolution investigations into patterns of gene loss along ancestral branches and their associations with geological events.

### Drift in anemia-associated genetic regions followed the decline in global temperatures

**Fig 1:**
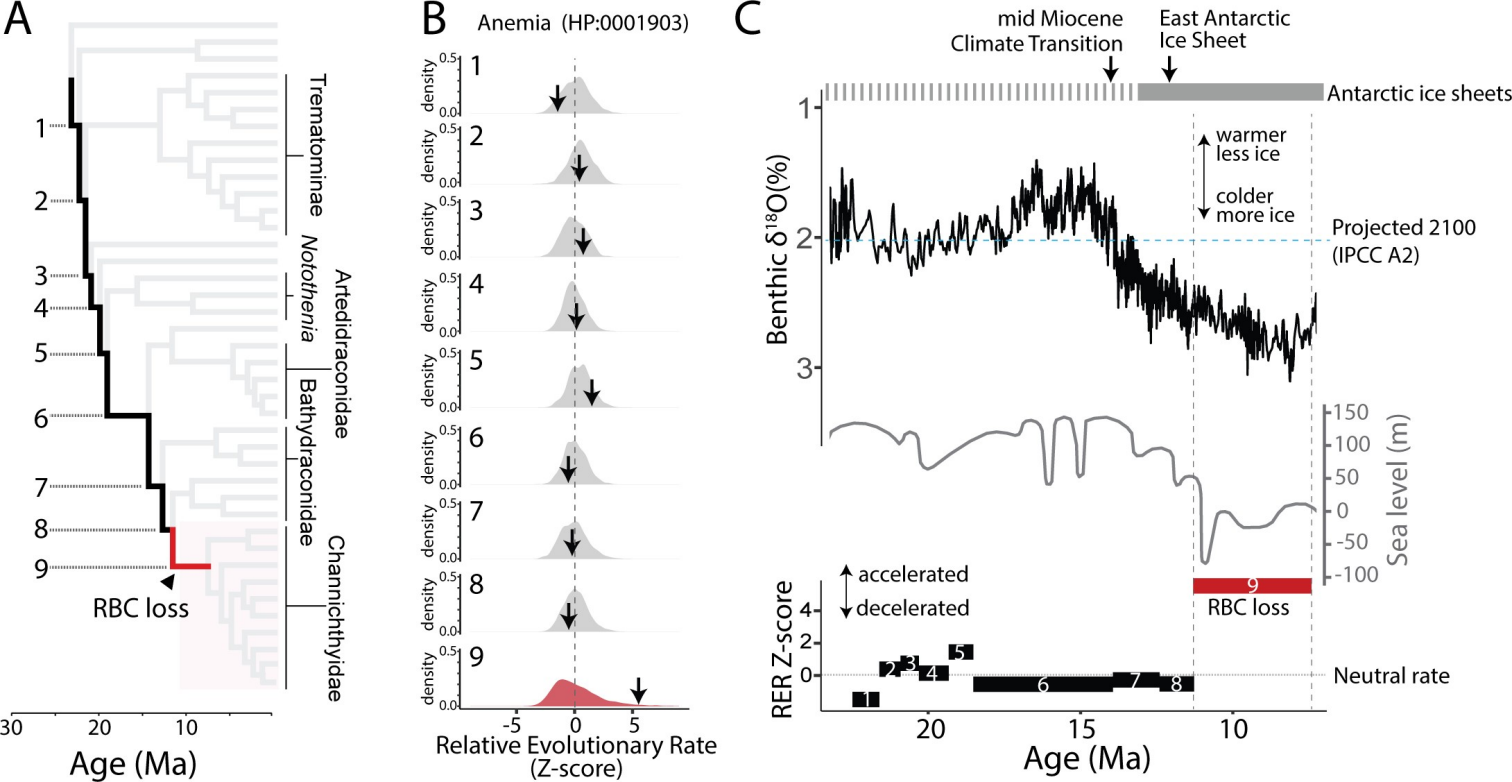
Drift in anemia-associated CNEs followed erythrocyte loss and decline in global temperatures. (**A**) Phylogeny of cryonotothenioids, highlighting the ancestral branches leading up to the loss of red blood cells (RBC) in icefishes (Channichthyidae). Numbers label branches in panel **B** and in the relative evolutionary rate (RER) plot of **C**. (**B**) Elevated RER of CNEs following loss of RBCs in icefishes. Distribution of Z-scores for average RER across groupings of conserved non-coding elements (CNEs). CNEs were linked to neighboring genes via the 'GREAT' algorithm [20] and then clustered based on the Human Phenotype Ontology (HPO) [19]. Z-scores > 0 are considered accelerated, while those < 0 have constrained evolution relative to the genome average. Arrows indicate positions in the histograms for the Anemia HPO term (HP:0001903). (**C**) RER increased in icefishes following loss of RBCs and the fall of global temperatures. The line numbers and lengths on the RER plot correspond to the branch labels and branch lengths on the time-calibrated phylogeny in **A**. The five-point moving average of global benthic δ^18^O ratios is adapted from Zachos *et al*. [21] and sea-level estimations from Haq *et al*. [22].

An important assumption underlying our understanding of icefish evolution is the link between evolved character states and cooling of the SO. Our dataset permitted us to directly test these associations. To track patterns of genome evolution associated with environmental change, we integrated paleoclimate data with a time-calibrated phylogeny of notothenioids. We determined evolutionary dynamics across phyletic branches ancestral to icefishes to identify changes in selection across climatic and evolutionary history. Protein coding genes were grouped into clusters of similar function based on the Human Phenotype Ontology [19], and CNEs were assigned to adjacent genes using the 'GREAT' algorithm [20]. We detected a significant enrichment for accelerated evolutionary rates in anemia-associated genetic regions coincident with the loss of erythrocytes on the branch leading to the common ancestor of icefishes (**Fig 1A** and **1B**, **S1 Table**). Notably, this trend was found for CNEs but not for coding sequences (**S2 Fig**, **S2 Table**), revealing a bias toward drift in putative gene-regulatory regions. Relaxation of purifying selection in anemia-associated regions was not observed prior to erythrocyte loss in the phylogeny (**Fig 1B** and **1C**).

**Fig 1C** shows that relaxation of purifying selection on CNEs near anemia-associated genes in icefishes followed pronounced global cooling, decreases in sea level, and the formation of stable Antarctic ice sheets after the mid-Miocene climate transition (MMCT) 14 Ma [21–23]. Prior to and during the MMCT, regional sea surface temperature (SST) estimates and other oceanic temperature proxies [23–28] exceeded the critical thermal maxima (CT_max_) of two extant icefish species [*Chaenocephalus aceratus* (13.9° ± 0.4°C); *Chionodraco rastrospinosus* (13.3° ± 0.2°C)] (**S3 Fig**), which are considered to be determined by the oxygen-carrying capacity of blood [29]. Therefore, evolution of the erythrocyte-null phenotype of Antarctic icefishes is tightly coupled, via physiology, to environmental cooling after the MMCT, in striking contrast to the increase in genetic diversity and positive selection for reduced skeletal density that evolved prior to the cryonotothenioid radiation [8].

### Correlation between the modern environment and relative evolutionary rate in notothenioids

Icefishes cohabit the frigid SO with several red-blooded notothenioid lineages. Although the cryonotothenioid radiation began ~22 Ma [30,31], the stably-cold temperatures and high oxygen concentrations of the SO necessary to facilitate viable reduction in hematocrit emerged well after the initial divergence of the group (**Fig 1C**, **S3 Fig**). Therefore, we propose that reduced hematocrits and tolerance of experimental anemia in several lineages of red-blooded notothenioids evolved independently of the icefish phenotypes [5,6].

**Fig 2:**
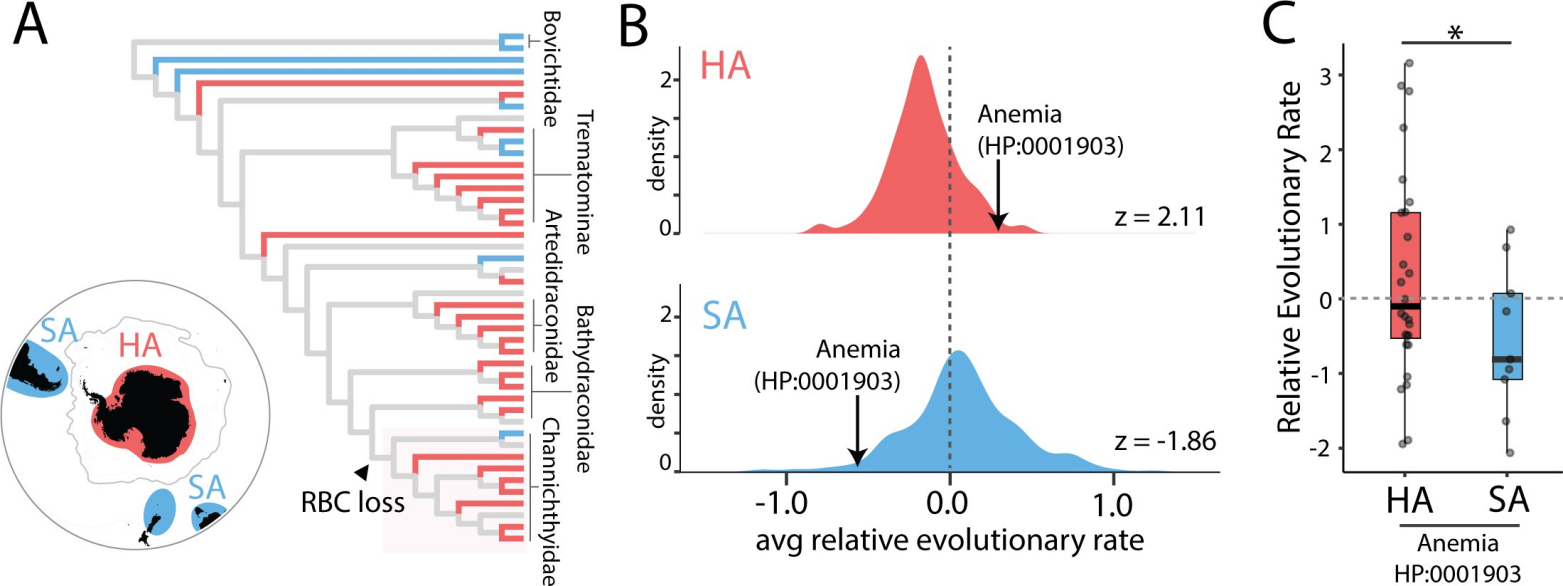
Elevated evolutionary rates in anemia-associated CNEs in high latitude notothenioids. (**A**) Notothenioid lineages designated as high-latitude Antarctic (HA) or sub-Antarctic (SA) as in Dornburg *et al*. 2017 [31]. (**B**) Distribution of average relative evolutionary rates across CNEs in all extant branches for each Human Phenotype Ontology term associated with at least 1000 CNEs. (**C**) Relative evolutionary rates of extant lineages. The asterisk indicates one-tailed t-test p-value < 0.05.

To evaluate drift in anemia-associated genetic regions among extant notothenioids, we compared relative evolutionary rates both in high-latitude Antarctic (HA) and in sub-Antarctic (SA) notothenioids, as recently compiled by Dornburg *et al* [31] (**Fig 2A**). Consistent with the expectation that lower temperatures reduced selective pressure on erythrocyte-associated regions, we found that HA, but not SA, notothenioids showed a significant bias toward elevated evolutionary rate in CNEs (**Fig 2B** and **2C**). This signal was largely driven by the icefishes, but also included several red-blooded notothenioid species (**S3 Table**). As a control, random selections of CNEs produced no deviation from neutral evolution when aggregated across these species’ ensembles (**S4 Fig**). Thus, relative evolutionary rate of anemia-associated CNEs correlated with latitude in extant notothenioids, which suggests that independent weakening of purifying selection on the erythroid genetic program is ongoing.

### Independent deterioration of erythrocyte-associated genes and occurrence of spherocytic anemia in cryonotothenioids

Given the decreased hematocrits, reduced hemoglobin oxygen affinities, and apparent relaxation of purifying selection at anemia-associated genetic regions in Antarctic notothenioids, we investigated whether deleterious mutations had accumulated in erythroid genes across the notothenioid phylogeny. We searched for deleterious mutations within a set of candidate genes involved in many facets of red blood cell development and function, including genes encoding cytoskeletal proteins (7 genes; e.g., *sptb* [32,33]), membrane and solute transporters (10 genes; e.g., *slc4a1a* [34]), carbonic anhydrases (6 genes; e.g., *car2* [35]), heme and hemoglobin biosynthesis-associated proteins (16 genes; e.g., *alas2* [36]) and transcription factors that regulate erythropoiesis (32 genes; e.g., *gata1* [37,38]) (**S4 Table**). Due to ambiguity in assigning function to missense variants, we focused our analysis on truncating variants (frameshifts, premature termination codons and whole gene deletions) and on missense SNPs previously identified at orthologous sites of genetic variation in human patients.

**Fig 3:**
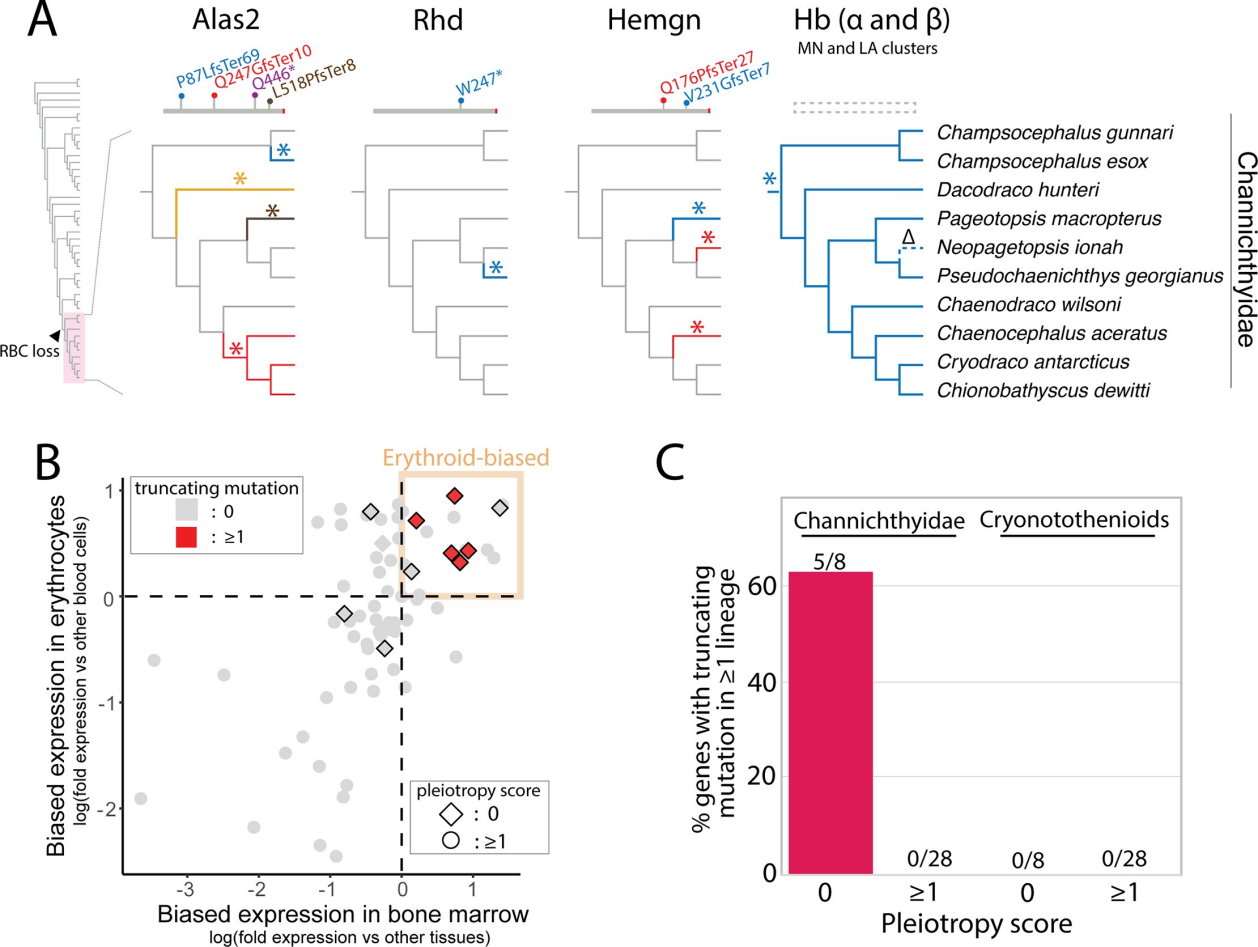
Truncating mutations in erythrocyte-associated genes. (**A**) Genes with truncating mutations or whole gene deletion in at least one icefish lineage. Asterisks indicate independent mutation events, with color corresponding to the allele above the cladogram. Δ indicates partial deletion of Hb in *N*. *ionah* with pseudogenization. See **S5–S7 Figs** for more detail on the Alas2, RhD and Hemgn mutations. See references 8–10 for analyses of the globin mutations. Arrow in **A** indicates position of red-blood cell (RBC) loss in icefishes (Channichthyidae) (**B**) Analysis of pleiotropy in erythroid genes (**S4 Table**). Genes were sorted by relative expression in mammalian erythrocytes vs other hematopoietic lineages and in mammalian bone marrow vs other organ systems. Genes were assigned a pleiotropy score ≥ 1 if mutations in these genes affect organ systems other than the hematopoietic system in the Mammalian Phenotype Ontology [74]. (**C**) Percentage of all erythroid-biased genes (**S5 Table**) with loss-of-function mutations in at least one lineage in icefishes (Channichthyidae) compared to other Antarctic notothenioid (Cryonotothenioids), showing enrichment for loss-of-function mutations in this gene set in species lacking red blood cells.

Results show the truncating variants in icefish erythrocyte genes appear to have evolved independently on multiple occasions. Truncating mutations in our candidate gene set were confined/unique to the icefishes (**Fig 3A**, **S5–S7 Figs**) and absent in other notothenioid clades. Nonsense mutations or frameshifts in *alas2* (erythroid-specific isoform) that are predicted to lead to premature termination were found in six of the 10 icefish species examined and arose independently in four lineages (**Fig 3A, S5 Fig**). Truncating mutations in *hemogen* (*hemgn*), which encodes an erythroid transcription factor [39,40], occurred independently in three icefish species, though many icefish species share a large deletion in this gene (**Fig 3A, S6 Fig**). Furthermore, *Rhd*, which encodes a blood group antigen, was truncated in *Pseudochaenichthys georgianus* (**Fig 3A, S7 Fig**). Consistent with our prior work, *globin* genes (*hba* and *hbb*) were also absent from most icefish species, with the exception of *Neopagetopsis ionah*, whose genome retained a pseudogenized version of the *globins* of the LA cluster (**Fig 3A**)[8,10].

**Fig 4:**
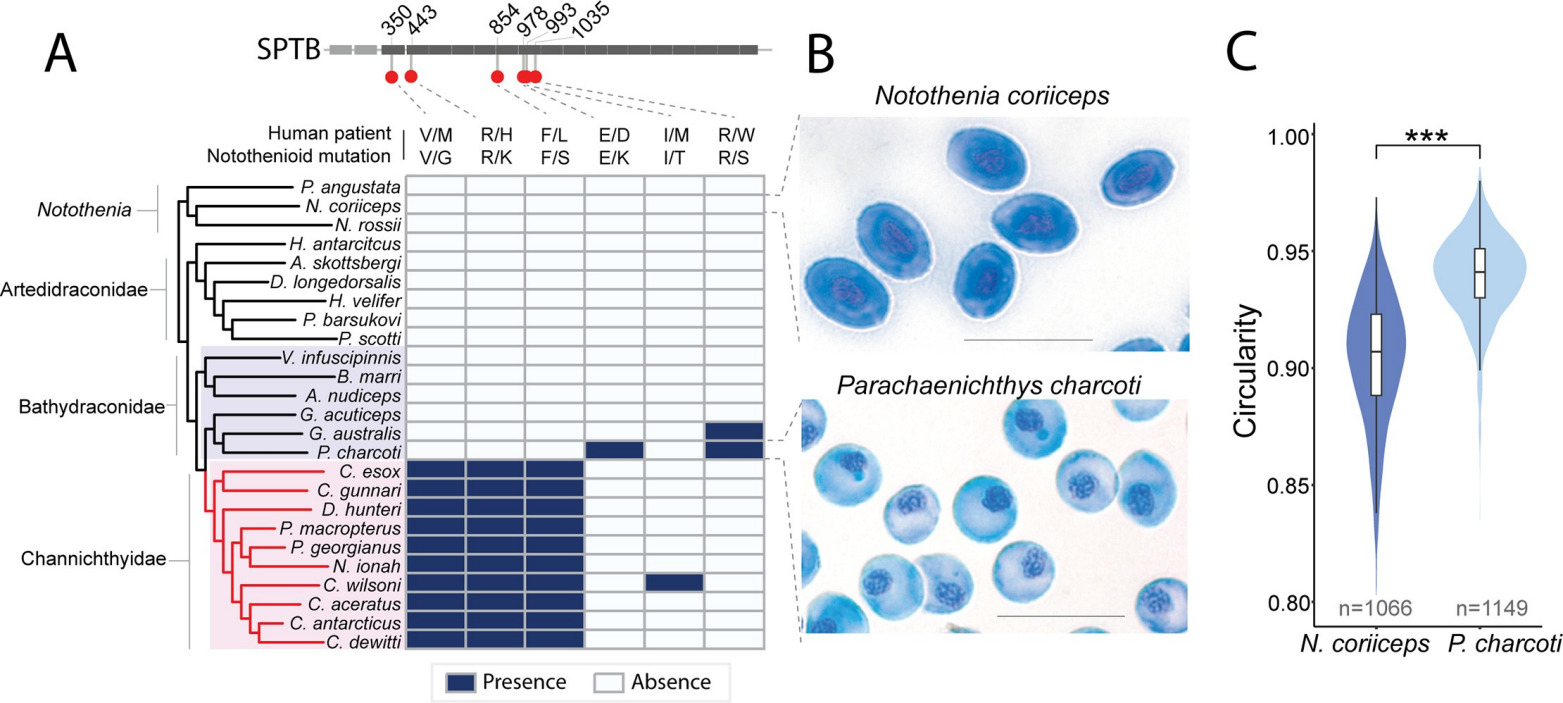
Clinically relevant variation in the notothenioid *beta spectrin* gene predicts convergent phenotypes of anemia between humans and Antarctic fishes. (**A**) Sites of mutation in human patients with hereditary spherocytosis/elliptocytosis that also have mutations in the notothenioid ortholog of *sptb*. Blue in heatmap indicates presence of the allele, white the absence. See **S8 Fig** for multiple sequence alignments. (**B**) Giemsa staining of peripheral blood revealed spherocytosis in erythrocytes of Charcot’s dragonfish (*P*. *charcoti*) compared to the red-blooded bullhead notothen (*N*. *coriiceps*). Scale bar = 20 μm. C) Circularity of erythrocytes in *N*. *coriiceps* and *P*. *charcoti* (*** Wilcoxon signed-rank test p-value < 2.2e^-16^). n indicates number of cells measured.

The *sptb* (*beta-spectrin*) locus is particularly informative with respect to the timing of evolutionary decay of the erythroid program in cryonotothenioids. Sptb (erythrocytic Beta spectrin) is a cytoskeletal protein that interacts with ankyrin and other proteins to organize the erythrocyte membrane and maintain the oval shape of the red cell [32,41]. Multiple mutations in human *SPTB* disrupt the erythrocyte cytoskeleton and cause hereditary elliptocytosis or spherocytosis, which are characterized by elliptical and/or spherical erythrocytes [33]. Ten icefishes (of 10 examined) evolved variants at three highly conserved and clinically relevant amino acid positions (**Fig 4A, S8 Fig**). In contrast, two dragonfish species (of six examined), *Parachaenichthys charcoti* and *Gerlachea australis*, accumulated missense mutations in *sptb* at *different* sites (**Fig 4A, S8 Fig**) that also correspond to human *SPTB* mutations [42]. Note, because our dataset involved analysis of pools of individuals, these mutations are presumed to be fixed in the species. Given that the decrease in SO temperatures followed the divergence of the icefish and dragonfish clades (**Fig 1, S3 Fig**), their distinct *sptb* mutations must have arisen by independent decay (**Fig 4A, S8 Fig**). Nonetheless, dragonfishes are the sister taxon to the white-blooded icefishes, and the two clades may share physiological and genetic contexts that predispose the loss of red cell function and production.

To determine whether misshapen erythrocytes were present in dragonfishes with *sptb* mutations, we analyzed blood smears of *P*. *charcoti* with Wright/Giemsa stain. We did not examine icefish blood smears due to the absence of mature red blood cells in peripheral blood. Intriguingly, we found that *P*. *charcoti* has spherical erythrocytes (**Fig 4B and 4C**), the same pathology described in human patients with mutations at the same positions [42], whereas *N*. *coriiceps*, which does not share variation at human patient sites in *sptb* gene, possessed oval erythrocytes. Together, the genetic and morphological evidence is consistent with the independent decay of the erythroid developmental program in dragonfishes and icefishes.

Outside of our candidate erythrocyte list, we found a few truncating variants in red-blooded notothenioids in non-erythrocyte oxygen-associated genes. This includes a truncating frameshift in the myoglobin of the barbled plunderfish *Artedidraco skottsbergi* and a truncation in the hemoglobin scavenging protein *haptoglobin* (*hp*) in the spiny plunderfish *Harpagifer anatarcticus* (**S9 and S10 Figs**).

### Pleiotropy shaped patterns of gene evolution following loss of erythrocytes by icefishes

Patterns of drift in anemia-associated genetic regions in our erythroid dataset were found largely in CNEs rather than within coding sequences (**Figs 1** and **2**). We hypothesize that pleiotropy acts to maintain a core scaffold of erythroid genes, even in the erythrocyte-null icefishes. To test this hypothesis, we developed a pleiotropy score for cryonotothenioid genes that integrates non-hematopoietic phenotypes and gene expression patterns based on mammalian functional annotation databases (see Materials and Methods). Low pleiotropy scores correspond to genes with predominantly erythroid phenotypes and expression.

Results showed that icefish genes with truncating mutations had low pleiotropy scores and highly erythroid-biased expression (*alpha-* and *beta-globins*, *rhd*, *alas2*, *hemgn*; **Fig 3B**, **S4 Table**). Furthermore, there was a statistically significant negative association between pleiotropy and the presence of deleterious mutations among all erythroid-biased genes (**Fig 3C**, **S5 Table**; Fisher’s exact test p-value = 0.0001). Thus, these classical “erythroid” genes are likely maintained in icefishes due to pleiotropic functions in other tissues.

### Patterns of mutation in CNEs predict the retention of erythroid progenitors in icefish blood

Erythrocytes develop from hematopoietic stem cells by specification of a myeloerythroid progenitor, commitment of the proerythroblast, and maturation through normoblast, reticulocyte, and terminal erythrocyte stages [43]. Given that icefishes apparently produce the full complement of myeloid and lymphoid lineages [17,18], two important questions emerge–what is (are) the stage(s) at which erythropoiesis fails, and what are the mechanism(s) underlying the failure? Because we detected specific signals of drift in icefish anemia-associated CNEs, we parsed patterns of drift in these regions across developmental specification of erythrocytes.

**Fig 5:**
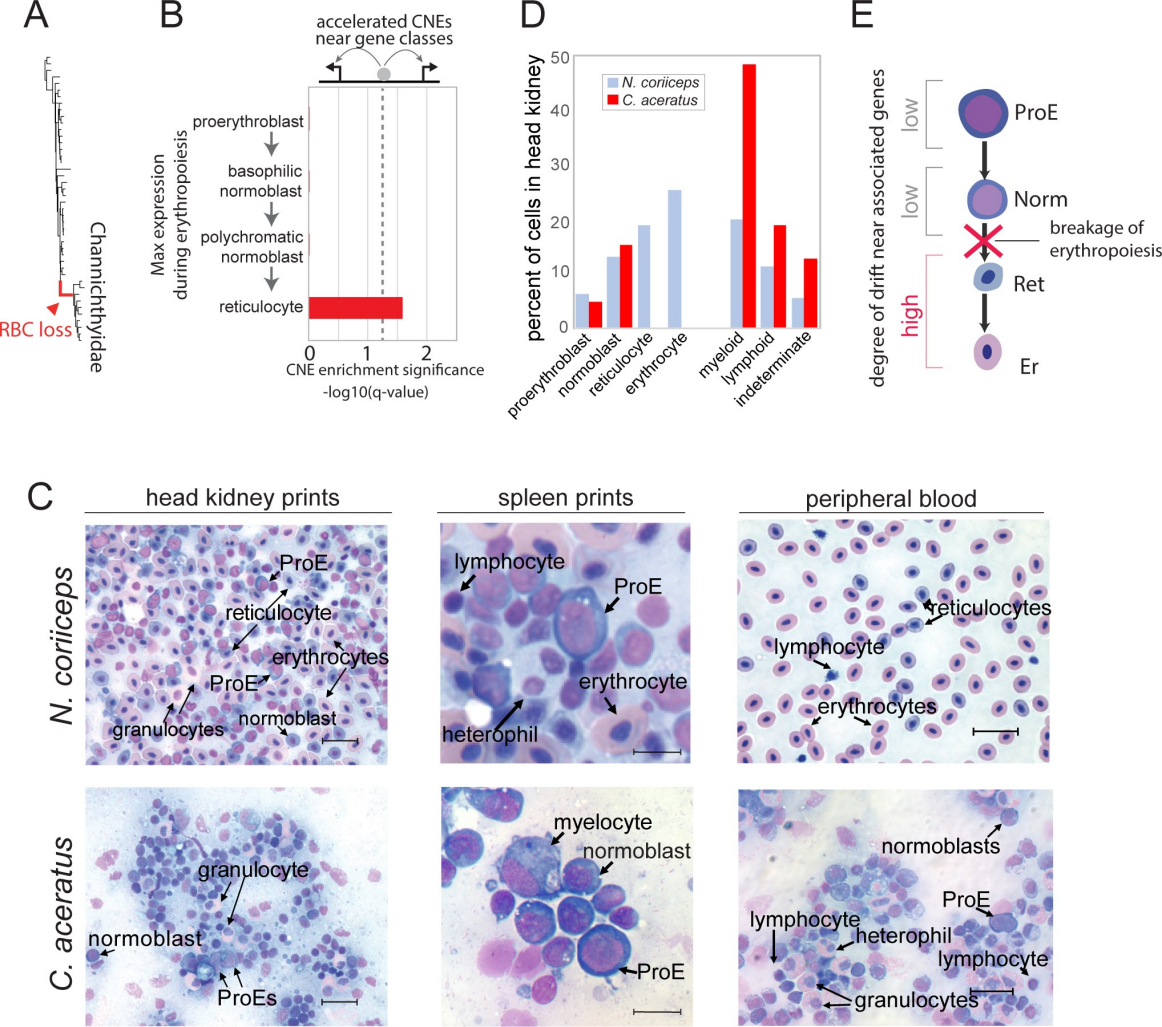
Patterns of accelerated sequence evolution in CNEs predict the presence of erythroid progenitors in icefish marrow and blood. (**A**) Branch leading to the common ancestor of icefishes from which the test for accelerated sequence evolution (phyloP) was run. (**B**) Enrichment for accelerated evolution of CNEs is biased toward genes that have maximum expression levels in reticulocytes. Data shown for the consensus gene list from primitive, fetal definitive, and adult definitive erythropoiesis in ErythronDB. See **S11 Fig** for individual breakdown of erythropoiesis types. (**C**) Prints of hematopoietic tissues (head kidney, spleen) and smears of peripheral blood from the “white-blooded” blackfin icefish *C*. *aceratus* and the red-blooded bullhead notothen *N*. *coriiceps* after staining with Wright/Giemsa. Both species possess erythroid progenitors (pro-erythroblasts (ProE)—circular cells with diameters ~15 μm, uncondensed nuclear chromatin, and densely blue-staining cytoplasm) and normoblasts (smaller proerythroblast derivatives with partial chromatin condensation and densely staining cytoplasm), but icefishes conspicuously lack reticulocytes (cells with an erythrocytic morphology but with a blue-staining cytoplasm due to high concentrations of globin and other mRNAs) and mature erythrocytes. Lymphoid and other myeloid lineages are present in both species. Scale bars: 25 μm in head kidney and peripheral blood, 10 μm in spleen. (**D**) Cell composition of head kidney prints from *N*. *coriiceps* (n = 1,420 cells) and the icefish *C*. *aceratus* (n = 825 cells). (**E**) Model of erythropoiesis in icefishes showing failure of differentiation/maturation (X) occurring at the normoblast (Norm) to reticulocyte (Ret) transition.

Using data from the murine ErythronDB to cluster genes by expression profile during erythrocyte maturation [44], we found that CNEs near genes that were highly expressed in reticulocytes had significantly elevated evolutionary rates on the ancestral branch leading to icefishes (**Fig 5A** and **5B**). This trend was observed across datasets for primitive, fetal definitive, and adult definitive erythropoiesis, and for the consensus gene set across all forms of erythropoiesis (**Fig 5A** and **5B**; **S11 Fig**). Given the conservation of CNEs near early-, but not late-, stage erythrocyte genes, we hypothesize that erythropoiesis in icefishes halts at the normoblast stages of late erythroid maturation, rather than at earlier stages, and that erythroid progenitors are present in icefish blood marrow.

To characterize hematopoietic lineages in notothenioid marrow, we examined head-kidney and spleen tissue prints (sites of leukopoiesis and erythropoiesis in fishes [43,45–47]) and peripheral blood smears from icefishes and red-blooded relatives (**Fig 5C** and **5D**; **S12 Fig**). **Fig 5C** and **5D** show that icefish marrow and peripheral blood possessed proerythroblasts and normoblasts but were devoid of reticulocytes or mature erythrocytes, whereas *N*. *coriiceps* marrow and blood contained the complete erythroid suite of cells. Furthermore, we also identified proerythroblasts and normoblasts in spleen prints from two other icefish species (*P*. *georgianus*, *C*. *rastrospinosus*; **S12 Fig**). In contrast, we found that the marrow and blood of icefishes and red-blooded notothenioids contained apparently similar distributions of leukocytes: lymphocytes, myelocytes, and granulocytes (**Fig 5C** and **5D**; **S12 Fig**). Taken together, these data indicate that erythropoiesis in icefishes fails during terminal maturation (**Fig 5E**).

## Discussion

Our results provide a comprehensive examination of the environmental, genetic, and developmental events involved in loss of erythrocytes in icefishes. By analyzing thousands of genes in a phylogenetic framework, we determined whether mutational events were shared among icefishes and other cryonotothenioids, and we used these patterns of mutation to predict natural phenotypes. Finally, we examined ancestral branches within the phylogeny to assess how changes to mutation rates aligned with paleoclimatic events.

As the global environment rapidly changes in response to anthropogenic impacts, understanding the mechanisms by which species have responded to past climate events is critically important. Here, we demonstrate an interaction between climate change, pleiotropy, and developmental constraint in shaping genome evolution. As many of the genes involved in erythropoiesis have pleiotropic roles in other tissues, the necessity of properly completing development appears to limit the allowable mutations that can occur in the genome–in this case most erythrocyte-associated genes remain largely intact. Thus, as species respond to climate change, this developmental constraint will play a role in shaping evolutionary trajectories.

### Erythrocyte loss: evolution in response to environmental change

Icefishes are the only vertebrate taxon whose species survive without red blood cells. How do species lose a cell type that is thought to be essential for viability? Through analysis of paleoclimate data and comparative genomics, we present a new perspective on the erythrocyte-null phenotype of icefishes. We find that loss of erythrocytes occurred following steep declines in global and local oceanic temperatures, which led to increased dissolved oxygen concentrations in the SO. Icefish genomes then evolved rapidly, with decay of erythrocyte-associated non-coding regions occurring only after the formation of stable Antarctic ice sheets. Thus, a proximal environmental trigger drove the dramatic changes in the erythroid genetic program of icefishes (and, independently, within the sister clade the dragonfishes). This is in striking contrast to the roles of standing genetic diversity and positive selection that underlie the reduced skeletal density that preceded the cryonotothenioid radiation [8].

### Erythrocyte loss: maladaptive or adaptive?

Loss of erythrocytes should present with strong negative selection against the resulting anemia. Montgomery and Clements [48] argue that the loss of red cells by icefishes represents a “disaptation”–“*an organismal character whose use to the organism is demonstrably inferior to that of a phylogenetically antecedent character*”–and that recovery via readaptation reduced the detrimental impact of ablation of the red cell. That the most recent common ancestor of the icefishes probably possessed the metabolic flexibility necessary to transition to an erythrocyte-null condition is demonstrated by the capacity of red-blooded notothenioids to survive poisoning of hemoglobin with CO [6] or severe anemia induced by phenylhydrazine [5]. Nevertheless, icefish blood has an oxygen carrying capacity <10% (by unit volume) that of red-blooded notothens [49]. Sidell and O’Brien [12] assert that numerous cardiovascular enhancements in icefishes, including increased vascular branching [12], enlargement of the heart [50,51], elevated mitochondrial densities in cells [52], and a four-fold increase in blood volume [53], were necessary to compensate for severe chronic anemia. As a result of these changes, an estimated 22% of resting metabolic rate in icefishes is devoted to cardiac function, compared to 0.5–5.0% in most temperate fishes [54,55]. Adding to the physiological calculus are mutations of key erythroid genes in the genomes of these fishes. We and others have identified loss-of-function alleles in the *alpha-* and *beta-globins* [8–10], *alas2*, *hemgn*, *haptoglobin* [16] and *myoglobin* [12] that have risen to fixation in some, if not all, icefish species. These mutations are likely to constrain the adaptive landscape of icefishes and to render impossible the re-evolution of erythroid function as the SO warms. Thus, one may argue that the energetic savings achieved by abrogation of red cell production are likely to be negated by the costs of the physiological compensations to overcome anemia and the constrained ability of icefishes to adapt to environmental fluctuations.

### Underlying genetic ‘scaffolding’ of traits and prediction of natural phenotypes

The peripheral blood of icefishes, like red-blooded notothenioids and other fishes, contains leukocytes, lymphocytes, heterophils/granulocytes, myelocytes and thrombocytes. Although we did not detect reticulocytes or mature erythrocytes in icefish blood, we found small numbers of proerythroblasts and normoblasts. In striking contrast, we find that proerythroblasts, and to a lesser extent normoblasts, are abundant in icefish marrow prints from pronephric (head) kidney and spleen (**Fig 5, S12 Fig**). Progenitor accumulation might result from blockage of terminal erythroid differentiation/maturation or from a futile physiological response to hypoxia and anemia. Failure to mature beyond the normoblast stage implies that the erythroid genetic program is compromised at a step critical for terminal differentiation. The mutational event(s) causing this disruption is (are) not yet known.

We identified strong signals in CNEs that led to the prediction that erythroid progenitors do exist in icefish hematopoietic tissues. The same analyses performed on coding sequences failed to identify or predict this phenotype. Thus, pleiotropy likely constrains the types of mutations that can occur following trait loss. Although drift is a major factor shaping icefish genomes and developmental programs of erythropoiesis, pleiotropy appears to have left much of the erythroid genetic pathway intact. Dollo’s Law argues that traits lost in a lineage do not re-evolve [56,57], and the nearly complete extinction of *globin* genes in icefishes makes the recovery of fully functional erythrocytes problematic. Nevertheless, the modest losses of key erythroid genes and the maintenance of erythroid progenitors might enable these species to ‘re-gain’ some aspects of red cell function.

### Evolutionary mutant models of human disease

Traits that are adaptive to diverse organisms in various environmental contexts are often maladaptive (i.e., pathological) in humans. There has been much interest in using evolutionary diversity to gain insights into human disease, including how species evolve to compensate for the deleterious aspects of certain traits [58–60]. Comparative trait analysis can be used to identify novel disease genes, as shown by analysis of gene expression in icefishes [39,61], or as a means of filtering Genome-Wide Association Study (GWAS) hits, in which a high percentage of associated loci lack an obvious functional mechanism [62]. Cryonotothenioids have numerous traits that phenocopy human diseases, including aglomerular kidneys, lipid accumulation, low skeletal density, mitochondrial proliferation, heart enlargement with spongy myocardium, and others [1,12]. Furthermore, some cryonotothenioid traits, such as reduced skeletal density, show enrichment for selection in human-disease loci [8]. Thus, comparative genomic analyses within the cryonotothenioids have the potential to power our understanding of human diseases.

As an example, we demonstrate in this report convergence in anemic phenotypes based on mutations in the *beta-spectrin* gene of Antarctic icefishes, dragonfishes, and humans. Not only do dragonfishes and human patients share spherical erythrocytes as a result of *beta-spectrin* mutations, but the mutations introduce amino acid substitutions at the same highly conserved positions. Furthermore, evolution of anemia appears to be ongoing in the sister taxa of the icefishes. We propose that icefishes and dragonfishes share genetic and physiological potentials to ameliorate the deleterious effects of anemia and that understanding this potential can be leveraged to treat the human disease.

## Materials and methods

### Ethics statement

The experimental use of notothenioid fishes was performed in accordance with protocol 18-0103R, which was approved by the Northeastern University Institutional Animal Care and Use Committee (IACUC).

### Notothenioid genomic datasets

We used our recently published dataset of a broad taxonomic sampling of 46 species of notothenioid fishes and close relatives, including *Percophis brasiliensis* as the sister taxon to notothenioids and *Percina caprodes* as an outgroup [8,63]. This dataset contains contigs constructed from cross-species targeted sequence enrichment for over 250,000 protein coding exons and conserved non-coding elements, with an average coverage of targeted regions >90% in all notothenioids (doi: 10.5281/zenodo.2628936).

### Multiple sequence alignment

Orthologous sequences within the dataset were mapped according to Daane *et al*. [8]. Non-coding sequences were aligned using Mafft v7.313 (parameters ‘*—maxiterate 1000—localpair—op 10—ep 10*’)[64]. For coding sequences, the frameshift-aware program MACSE v2.03 was used (parameters ‘*-prog alignSequences -seq -seq_lr -fs_lr 10 -stop_lr 15*’)[65]. The multiple sequence alignment was pruned using GUIDANCE v2.02 to mask residues with scores <0.6 (parameters ‘*—bootstrap 25—mafft—maxiterate 100*,*—localpair—op 10—ep 10’*) [66].

### Reconstruction of gene sequences

As in Daane *et al*. [8], we reconstructed full gene sequences from the contigs that represented individual constituent coding exons. Orthologous exons were identified in the *Gasterosteus aculeatus* (three-spine stickleback) reference genome through reciprocal BLAST. We concatenated single-copy exons in the same order as they appear in the *Gasterosteus aculeatus* reference genome. Transcripts containing isoforms were merged into a non-redundant gene sequence containing all possible exons. A total of 18,600 gene sequences were reconstructed for each species.

### CNE association with genes

Because enhancers can regulate gene expression for genes many kilo- and mega-bases away from transcription start sites, prediction of regulatory targets is difficult *in silico*. To infer potential *cis*-regulatory targets of the CNEs, and thus link CNEs to putative biological function, we assigned CNEs to neighboring genes using the Genomic Regions Enrichment of Annotations Tool ('GREAT')[20]. This approach links CNEs to the transcription start site of the nearest neighboring genes within specified windows (minimum basal window is 5 kb upstream and 1 kb downstream of transcription start sites, extended up to 1 Mb or until overlap with the basal window from another gene) while allowing overlap such that multiple genes can be associated with the same CNE. This approach has much higher statistical power for detecting gene ontology enrichment of CNEs when compared to simple distance-based approaches for associating CNEs to putative regulatory targets [20].

### Patterns of sequence evolution

Relative evolutionary rates were estimated using the program RERConverge (parameters ‘*transform = "sqrt"*, *weighted = T*, *scale = T*, *cutoff =* 0’)[67]. As long branches exhibit higher degrees of variance compared to short branches, RERconverge includes a heteroskedasticity correction that increases comparative statistical power across the phylogeny [68].

We also assessed accelerated sequence evolution along pre-specified ancestral branches using the program phyloP, as implemented in PHAST v1.4 (parameters ‘*—method LRT—no-prune—features—mode ACC’*)[69,70]. The tree model for phyloP was derived using phyloFit and the species tree [8]. CNE tree models were based on 2,912 elements ≥ 250 bp with ≥ 85% coverage in all species.

### Gene cluster enrichment

We grouped notothenioid genes into specific clusters using several databases of mammalian orthologs. Since many of the evolved phenotypes in notothenioids are comparable to human pathologies, we utilized the Human Phenotype Ontology database (downloaded April 2018). We further used groupings of genes according to gene expression profiles during erythropoiesis in ErythronDB [44,71]. Gene identifiers for both databases were converted to Ensembl gene IDs followed by conversion to stickleback identifiers using Ensembl Biomart [72].

For analysis of relative evolutionary rate across a gene cluster, Z-scores were generated for each term by comparing the mean relative evolutionary rate from all genes within a gene cluster to a random distribution of 1,500 bootstrap resamples of equivalent bin sizes. Z-scores were calculated using SciPy (stats.scipy).

We also assessed patterns of cumulative polygenic enrichment within each gene cluster using the SUMSTAT approach [73]. For phyloP, we normalized the distribution of log-likelihood ratio test values (ΔlnL) by taking the fourth root (ΔlnL4). The ΔlnL4 score was then summed for all genes within an ontology and an enrichment p-value was estimated from the empirical sum(ΔlnL4) score through bootstrap resampling (1,500 replicates).

In all enrichment analyses, p-values were corrected using FDR (Python module statsmodels v0.6.1; fdrcorrection0).

### Analysis of notothenioid gene mutations in human orthologs

As our data was already converted to stickleback orthologs (see 'Reconstruction of gene sequences'), we used Ensembl Biomart to map orthologs between stickleback and human annotations [72]. To identify the site of orthologous human mutations, we performed multiple sequence alignments of each translated exon using Mafft v7.313 (parameters '—*maxiterate* 1000—*localpair*—*op* 10—*ep* 10—*addfragments* '). To avoid generating inferences based on non-homologous sites, we only considered amino acid positions where the ancestral notothenioid and human amino acids were identical. We then used the ClinVar database to check for variants in human patients at sites of notothenioid mutation [42].

### Assessment of pleiotropy in coding regions

We developed a pleiotropy score based on the number of recorded non-hematopoietic system phenotypes for each gene in the Mammalian Phenotype Ontology (downloaded March 2019), which is a record of phenotypes in mouse mutants organized by organ and tissue system [74]. To calculate pleiotropy scores in non-blood tissues, we removed descendent ontologies within the "Hematopoietic System Phenotype" from each gene. We excluded indirect phenotypes, such as pallor, abnormal iron or blood chemistry, body or organ size, and spleen abnormalities (**S6 Table**), because they are secondary to reduction in hematocrit. A score of 0 indicates absence of non-hematopoietic system phenotypes, whereas a score ≥ 1 would indicate the presence of a phenotype outside of this system (e.g. craniofacial phenotype or muscle phenotype).

To complement the mouse phenotype data, we also included gene expression data to identify erythroid-biased genes. We used the Human Protein Atlas to distinguish between genes expressed throughout the body with those predominantly expressed in hematopoietic tissues (mammalian bone marrow)[75]. We further compared expression across multiple hematopoietic cell types to find erythroid-enriched genes (Array Express: E-MTAB-3079 on the Expression Atlas [76]).

Truncating mutations were indicated by the absence of read coverage across the gene and/or the presence of premature termination via frameshift or nonsense mutation. We required a minimum of three sequencing reads for any frameshift or nonsense mutation to be reported. Unless otherwise indicated, all frameshifts or nonsense mutations reported are fixed in our sequencing read data, which is pooled from populations of 5 or more individuals (see [8]).

### Tree calibration

We time-calibrated our species tree using TreePL (parameters ‘*smooth = 0*.*1*, *cv*, *randomcv*, *opt = 1 moredetail optad = 1*, *moredetailad*, *optcvad = 2*, *moredetailcvad*, *thorough*’)[77]. We used date priors from two recent time-calibrated notothenioid phylogenies [30,31]. The minimum and maximum age estimate priors for the most recent common ancestor (MRCA) were: *Pseudaphritis* + Eleginopsioidea (62.5–87.1 Ma), *Harpagifer*-*Pogonophryne* (7.7–13.0 Ma), *Bathydraco*-*Chaenocephalus* (9.4–13.3 Ma), *Notothenia* (15.2–20.5 Ma), Cryonotothenioidea (18.6–23.9 Ma), Eleginopsiodea (37.2–53.2 Ma).

### Analysis of notothenioid blood

Three species of channichthyids (*Chaenocephalus aceratus*, *Pseudochaenichthys georgianus*, and *Chionodraco rastrospinosus*), two species of nototheniids (*Notothenia coriiceps* and *Gobionotothen gibberifrons*), and a single dragonfish species (*Parachaenichthys charcoti*) were collected by bottom trawling from the *R/V Polar Duke* or the *R/V Laurence M*. *Gould* near Low and Brabant Islands in the Palmer Archipelago. The fish were transported alive to Palmer Station, Antarctica, where they were maintained in seawater aquaria at -1.5°C to +1.0°C.

Whole blood (5–25 ml) was collected from live fishes via caudal venipuncture using heparinized syringes. Aliquots (~5–10 μl) from red-blooded species were directly smeared on glass microscope slides by standard techniques [78]. Because icefish blood contains ~4% cells by volume, cells were concentrated by low-speed centrifugation of 5 or 10 ml aliquots (clinical centrifuge, 1000 rpm, 5 min, room temperature), and pellets were resuspended in 0.5 ml Notothenioid Ringer’s solution (260 mM NaCl, 5 mM KCl, 2.5 mM MgCl_2_, 2.5 mM CaCl_2_, 2 mM NaHCO_3_, 2 mM NaH_2_PO_4_, 5 mM glucose) on ice before blood smears (~5–10 μl) were prepared. Head kidney and spleen tissues were dissected from euthanized fish, and prints were prepared by pressing each tissue gently onto microscope slides to deposit a monolayer of cells. Cells of smears and prints were then fixed in 100% methanol for 5 min.

Blood smears and tissue prints were stained with Wright’s solution (0.1% w/v, pH = 6.8; Sigma-Aldrich) for 15 s, washed for 1 min in distilled water, and then stained with Giemsa solution (0.4% w/v, pH = 7.2, Sigma-Aldrich) for 1.5 min. Slides were then washed for 3 min in distilled water and air-dried. Wright’s stains the cytoplasm light blue, and Giemsa stains the nucleus a deeper blue/purple with collagen and other tissue elements staining pink to rose [79]. Micrographs were recorded using a Nikon E800 microscope equipped with differential interference contrast optics, a SPOT 7.2 Color Mosaic CCD camera (Diagnostic Instruments, Inc.), and SPOT 5.1 imaging software.

### Quantitation of erythrocyte morphology

Erythrocyte morphology was determined using Fiji [80]. Wright/Giemsa-stained peripheral blood smears from one individual of *N*. *coriiceps* and one of *P*. *charcoti* were quantified. To smooth edges and reduce background noise, a Gaussian blur (sigma = 1) and rolling ball background subtraction (rolling = 7) was applied to each image of a field of cells. Image contrast was enhanced ("saturated = 0.1 normalize"), and the image was converted to a binary through Auto-Thresholding ("method = Minimum"). Cells were further smoothed and gaps filled through opening and closing operations and the "fill holes" command. To ensure accurate measurements of cell shape, we ignored particles with unusual morphologies that may have been artifacts of automated thresholding. We also excluded cells that touched other cells or the edge of the frame by restricting particle size to an area of 70–150 μm^2^ and by removing particles with circularity < 0.80 and solidity < 0.93. We analyzed 1,066 cells for *N*. *coriiceps* and 1,149 cells for *P*. *charcoti* for circularity (C = 4πArea/Perimeter^2^).

## Supporting information

S1 Fig Phylogeny of notothenioid species included in this study. Tree topology from Daane *et al*. [8]. Phylogenetic relationships inferred from ASTRAL using 11,627 gene trees. All nodes in the phylogeny are supported by 100% quadpartition posterior probability. Asterisk (*) indicates position of red blood cell loss in the icefishes (Channichthyidae).
(TIF)

S2 Fig Drift in coding sequences of anemia-associated genes did not follow erythrocyte loss or the decline in global temperatures. (**A**) Phylogeny of cryonotothenioids, highlighting the ancestral branches leading up to the loss of red blood cells (RBC) in icefishes (Channichthyidae). Numbers label branches in panels **B** and **C**. (**B**) Elevated relative evolutionary rate (RER) following loss of RBCs in icefishes. Distribution of Z-scores for average RER across groupings of genes. These genes were then clustered based on the Human Phenotype Ontology (HPO) [19]. Arrow indicates position in histogram of the Anemia HPO term (HP:0001903). Z-scores > 0 are considered accelerated, while those < 0 have constrained evolution relative to the genome average. (**C**) Relative evolutionary rate across genes in icefishes following loss of RBCs and the fall of global temperatures remained steady. The five-point moving average of benthic δ18O ratios is adapted from Zachos et al. 2001 [21] and sea level estimations from Haq et al. 1987 [22].
(TIF)

S3 Fig Global and local paleo-temperature estimates and the loss of erythrocytes in icefishes. Overlay of time-calibrated phylogeny of cryonotothenioids and paleoclimate estimates shows loss of red blood cells (*, red branch) following decreases in global and local temperatures. (**A**) Sea surface temperature (SST) reconstructions from multiple Southern Ocean drill sites. Site location, SST method and citation are indicated in the inset. Modern and paleo drill site locations adapted from Hartman *et al*., 2018 [25], and mapped using the Ocean Drilling Stratigraphic Network Plate Tectonic Reconstruction Service (http://www.odsn.de/odsn/services/paleomap/paleomap.html). CT_max_ for the blackfin icefish, *Chaenocephalus aceratus*, is indicated by the dashed line. (**B**) The five-point moving average of global benthic δ^18^O ratios is adapted from Zachos *et al*. 2001 [21]. Higher δ^18^O ratios indicate colder temperatures and more ice.
(TIF)

S4 Fig Enrichment for elevated evolutionary rate in anemia-associated genetic regions compared to random gene sets. Three random sets of genes equal to the number of genes in HP:0001903 (n = 360) were created and the relative evolutionary rate between species distributed in the high-Antarctic (HA) and sub-Antarctic (SA) were compared. * indicates one-tailed t-test p-value < 0.05; n.s. is not significant.
(TIF)

S5 Fig Truncating mutations identified in icefish erythroid-specific 5-aminolevulinate synthase (alas2) gene. (**A**) Notothenioid phylogeny showing presence of truncating alleles (*) in four icefish species. (**B**) Mutant alleles; asterisk color corresponds to branches in **A**. (**C**) Sequencing read depth for each species aligned to the *Notothenia coriiceps* reference genome. Gaps in read depth correspond to deletions in each read relative to the reference genome. (**D**-**G**) The icefishes show distinct frameshifts and truncations in Alas2 compared to the *N*. *coriiceps* reference sequence. Alignment start/stop coordinates in **D**-**G** are based on position in the *N*. *coriiceps* genome assembly (XP_010782407.1).
(TIF)

S6 Fig Truncating mutations identified in icefish hemogen gene. (**A**) Phylogeny of the notothenioids showing the presence of truncating alleles (*) in three icefish species. (**B**) Mutant alleles; asterisk color corresponds to branches in **A**. (**C**) Sequencing read depth for each species aligned to the *Notothenia coriiceps* reference genome. Gaps in read depth correspond to deletions in each read relative to the reference genome. (**D**) *Chaenocephalus aceratus* and *Neopagetopsis ionah* show identical frameshifts and truncations in Hemgn compared to the *N*. *coriiceps* reference. (**E**) *Pageotopsis macropterus* shows a different frameshift and truncation. Alignment start/stop coordinates in **D** and **E** are based on position in the *N*. *coriiceps* genome assembly (XP_010773828.1).
(TIF)

S7 Fig Truncating mutation identified in Pseudochaenichthys georgianus Rh blood group D antigen (rhd) gene. (**A**) Notothenioid phylogeny showing presence of a truncating allele in *P*. *georgianus* (*). (**B**) The mutation encoded by the allele. (**C**) Sequencing read depth for *P*. *georgianus* as aligned to the *Notothenia coriiceps* reference genome. The gap in read depth corresponds to a deletion in each read relative to the reference genome. (**D**) *P*. *georgianus* shows a frameshift and truncation in Rhd compared to the *N*. *coriiceps* reference sequence. Alignment start/stop coordinates are based on position in the *N*. *coriiceps* genome assembly (XP_010782194.1).
(TIF)

S8 Fig Dragonfish and icefish mutations at highly-conserved and clinically-relevant sites in Beta-spectrin. Variant amino acid substitutions in Beta-spectrin of the dragonfish *Parachaenichthys charcoti* and a representative icefish *Chaenodraco wilsoni* highlighted in red. Beta-spectrin sequences for three-spined stickleback (*Gasterosteus aculeatus*), spotted gar (*Lepisosteus oculatus*), elephant shark (*Gallorhinchus milii*) and human (*Homo sapiens*) are provided for comparison. The dbSNP identifier (ClinVar) for deleterious variants found in human patients with spherocytic anemia/elliptocytosis are shown above each alignment.
(TIF)

S9 Fig Truncating mutations identified in notothenioid myoglobin gene. (**A**) Phylogeny of the notothenioids showing the presence of truncating alleles (*) in three species. (**B**) Mutant alleles; asterisk color corresponds to branches in **A**. (**C**) Sequencing read depth for each species aligned to the *Notothenia coriiceps* reference genome. Gaps in read depth correspond to deletions in each read relative to the reference genome. (**D**) Red-blooded species *Artedidraco skottsbergi* Mb compared to the *N*. *coriiceps* reference. (**E**) *Champsocephalus gunnari* and *C*. *esox* shows identical frameshifts in Mb. Alignment start/stop coordinates in **D** and **E** are based on position in the *N*. *coriiceps* genome assembly (NP_001290223.1).
(TIF)

S10 Fig Truncating mutations identified in notothenioid haptoglobin gene. (**A**) Phylogeny of the notothenioids showing the presence of truncating alleles (*) in three species. (**B**) Mutant alleles; asterisk color corresponds to branches in **A**. (**C**) Sequencing read depth for each species aligned to the *Notothenia coriiceps* reference genome. Gaps in read depth correspond to deletions in each read relative to the reference genome. (**D**) Red-blooded species *Harpagifer antarcticus* Hp compared to the *N*. *coriiceps* reference. The icefish species (**E**) *Champsocephalus gunnari* and (**F**) *Dacodraco hunteri* have different frameshifts and truncations in Hp. Alignment start/stop coordinates in **D**-**F** are based on position in the *N*. *coriiceps* genome assembly (XP_010770321.1).
(TIF)

S11 Fig Enrichment for accelerated sequence evolution in conserved non-coding elements (CNEs) near genes that are maximally expressed at distinct stages of erythropoiesis. Three waves of mammalian erythropoiesis are defined by distinct patterns of gene expression and (locations): primitive (yolk sac blood island), fetal definitive (liver) and adult definitive (bone marrow). For each erythropoietic wave, accelerated evolution of CNEs near maximally expressed genes is shown for four cellular stages of erythroid differentiation/maturation: proerythroblast, basophilic erythroblast/normoblast, polychromatic erythroblast/normoblast, reticulocyte. The Consensus is the intersection of maximally expressed genes across each the three erythropoietic waves. Dashed line corresponds to q-value of 0.05. Gene expression data from ErythronDB [44].
(TIF)

S12 Fig Spleen prints from three notothenioid species: Wright/Giemsa-stained. Two “white-blooded” icefishes, *Pseudochaenichthys georgianus* and *Chionodraco rastrospinosus*, show the presence of erythroid progenitors [proerythroblasts (ProEs) and normoblasts] but lack later stages of maturation (e.g., reticulocytes, erythrocytes). By contrast, the red-blooded notothen, *Gobionotothen gibberifrons*, displays the complete erythropoietic progression: ProE → normoblast → reticulocyte → erythrocyte. Scale bar = 10 μm.
(TIF)

S1 Table Human phenotype ontology (HPO) enrichment of CNEs under accelerated sequence evolution. (XLSX)

S2 Table Human phenotype ontology (HPO) enrichment of coding sequences under accelerated sequence evolution. (XLSX)

S3 Table Relative evolutionary rate and notothenioid biogeography. (PDF)

S4 Table Coverage and mutations in candidate erythrocyte genes. (PDF)

S5 Table Coverage and mutations in erythroid-biased genes. (PDF)

S6 Table Excluded terms from Mammalian Phenotype Ontology (MP) in pleiotropy analysis. (PDF)
