# Supplementary figures and images for: Developmental constraint shaped genome evolution and erythrocyte loss in Antarctic fishes following paleoclimate change
Source: PLoS Genet. 2020 Oct 27;16(10):e1009173. doi: 10.1371/journal.pgen.1009173 (PMC7660546; doi:10.1371/journal.pgen.1009173)

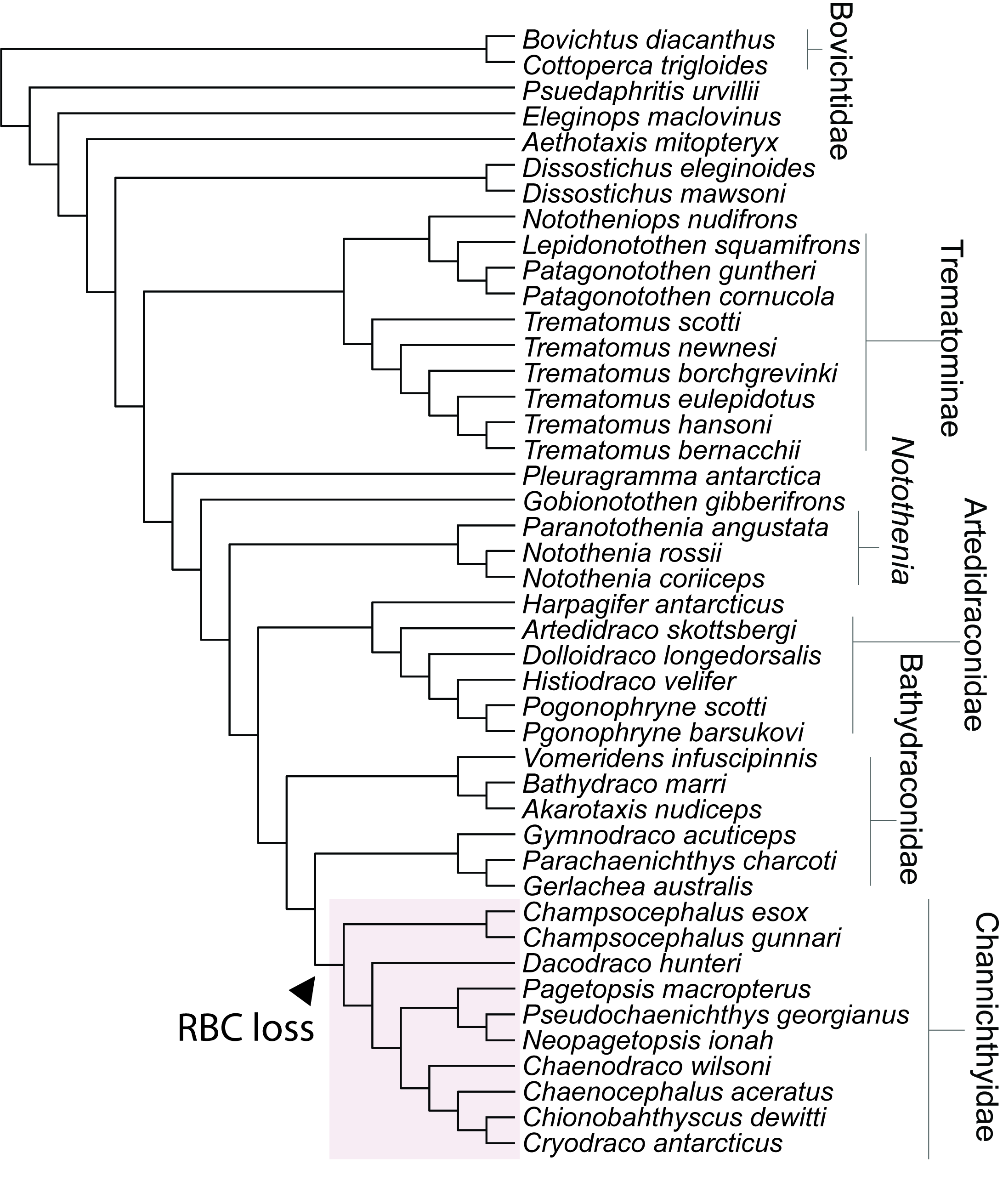

Supplement: S1 Fig — Tree topology from Daane et al. [8]. Phylogenetic relationships inferred from ASTRAL using 11,627 gene trees. All nodes in the phylogeny are supported by 100% quadpartition posterior probability. Asterisk (*) indicates position of red blood cell loss in the icefishes (Channichthyidae). (TIF) [file pgen.1009173.s001.tif]

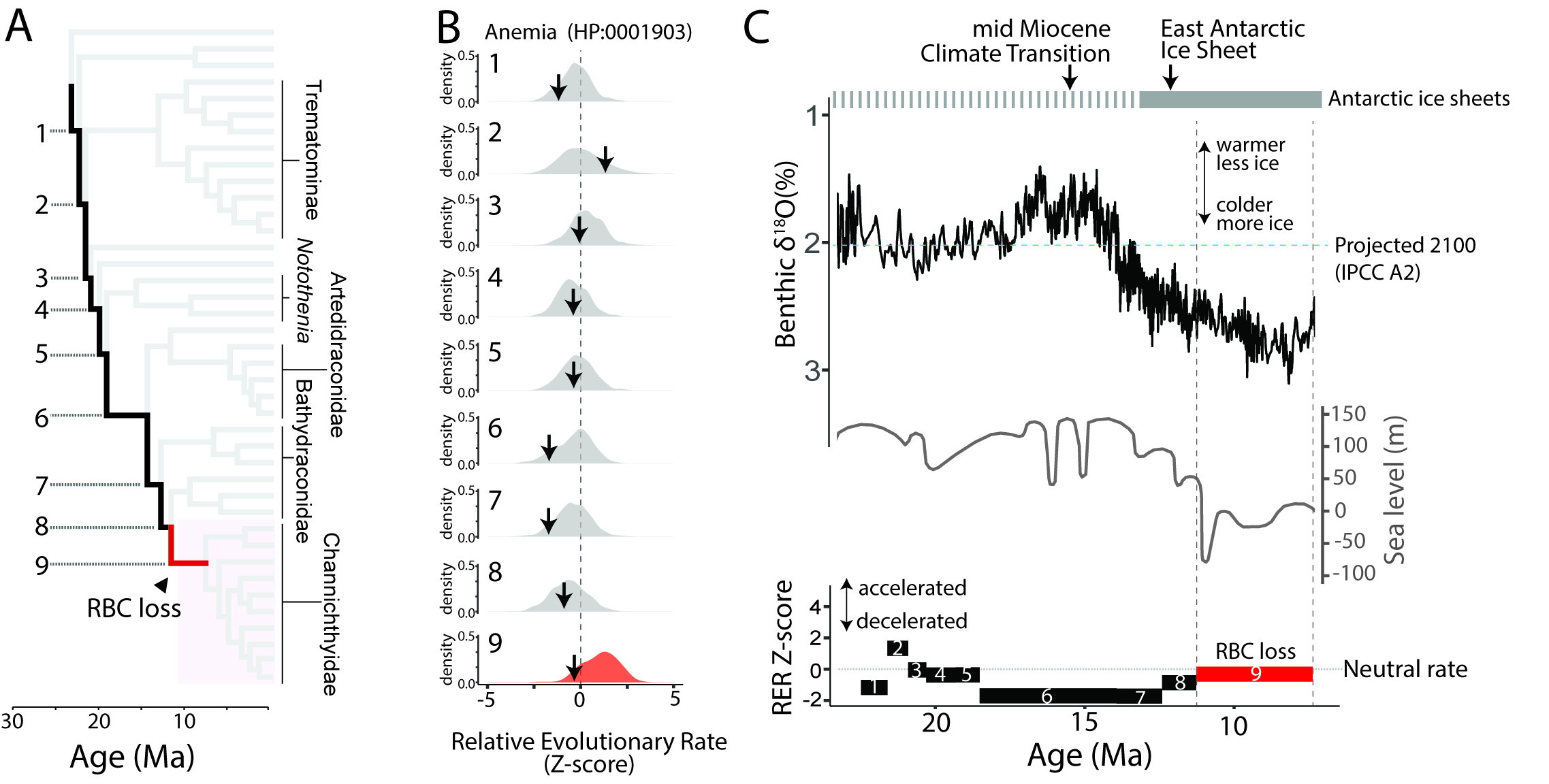

Supplement: S2 Fig — (A) Phylogeny of cryonotothenioids, highlighting the ancestral branches leading up to the loss of red blood cells (RBC) in icefishes (Channichthyidae). Numbers label branches in panels B and C. (B) Elevated relative evolutionary rate (RER) following loss of RBCs in icefishes. Distribution of Z-scores for average RER across groupings of genes. These genes were then clustered based on the Human Phenotype Ontology (HPO) [19]. Arrow indicates position in histogram of the Anemia HPO term (HP:0001903). Z-scores > 0 are considered accelerated, while those < 0 have constrained evolution relative to the genome average. (C) Relative evolutionary rate across genes in icefishes following loss of RBCs and the fall of global temperatures remained steady. The five-point moving average of benthic δ18O ratios is adapted from Zachos et al. 2001 [21] and sea level estimations from Haq et al. 1987 [22]. (TIF) [file pgen.1009173.s002.tif]

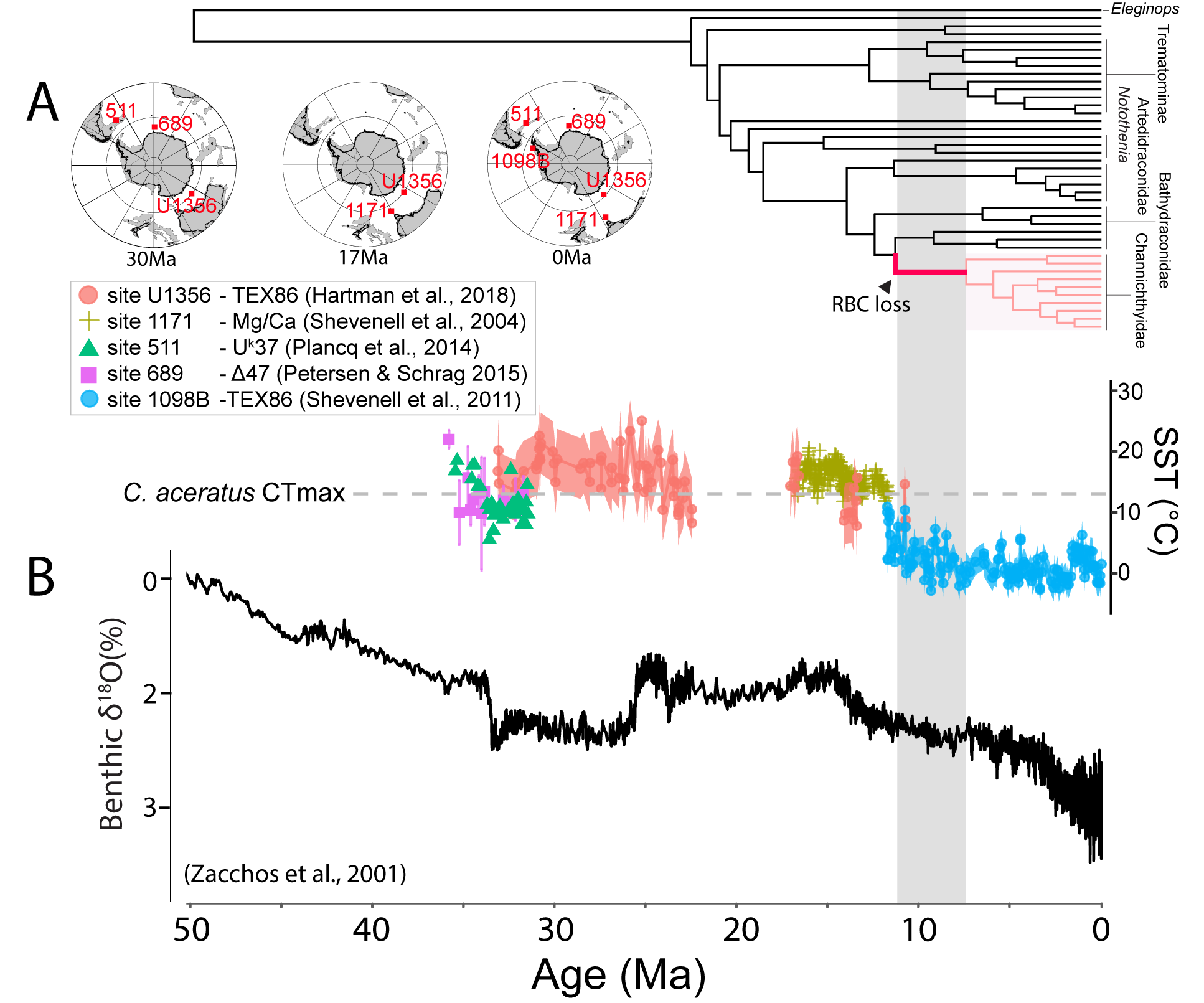

Supplement: S3 Fig — Overlay of time-calibrated phylogeny of cryonotothenioids and paleoclimate estimates shows loss of red blood cells (*, red branch) following decreases in global and local temperatures. (A) Sea surface temperature (SST) reconstructions from multiple Southern Ocean drill sites. Site location, SST method and citation are indicated in the inset. Modern and paleo drill site locations adapted from Hartman et al., 2018 [25], and mapped using the Ocean Drilling Stratigraphic Network Plate Tectonic Reconstruction Service (http://www.odsn.de/odsn/services/paleomap/paleomap.html). CTmax for the blackfin icefish, Chaenocephalus aceratus, is indicated by the dashed line. (B) The five-point moving average of global benthic δ18O ratios is adapted from Zachos et al. 2001 [21]. Higher δ18O ratios indicate colder temperatures and more ice. (TIF) [file pgen.1009173.s003.tif]

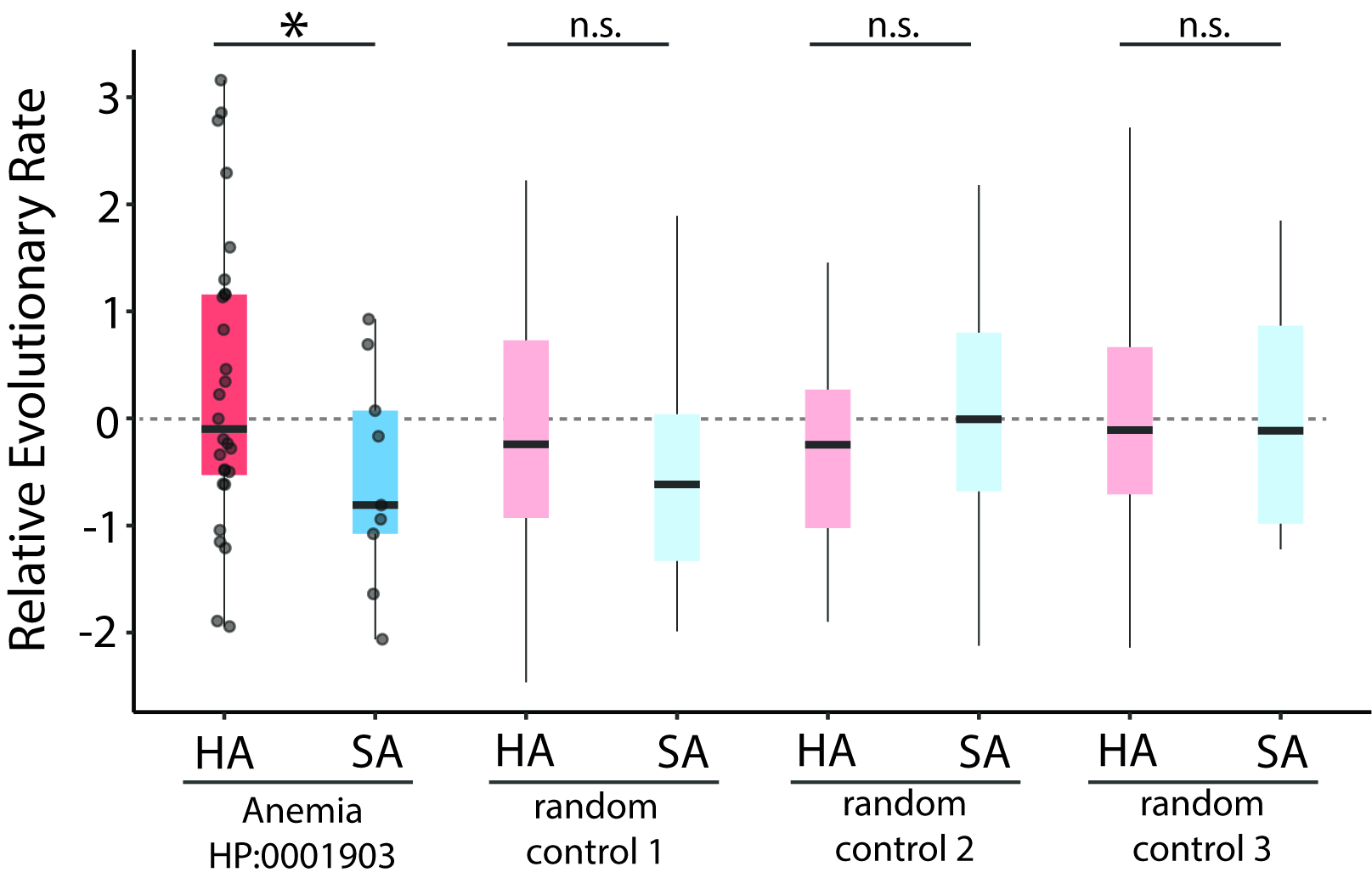

Supplement: S4 Fig — Three random sets of genes equal to the number of genes in HP:0001903 (n = 360) were created and the relative evolutionary rate between species distributed in the high-Antarctic (HA) and sub-Antarctic (SA) were compared. * indicates one-tailed t-test p-value < 0.05; n.s. is not significant. (TIF) [file pgen.1009173.s004.tif]

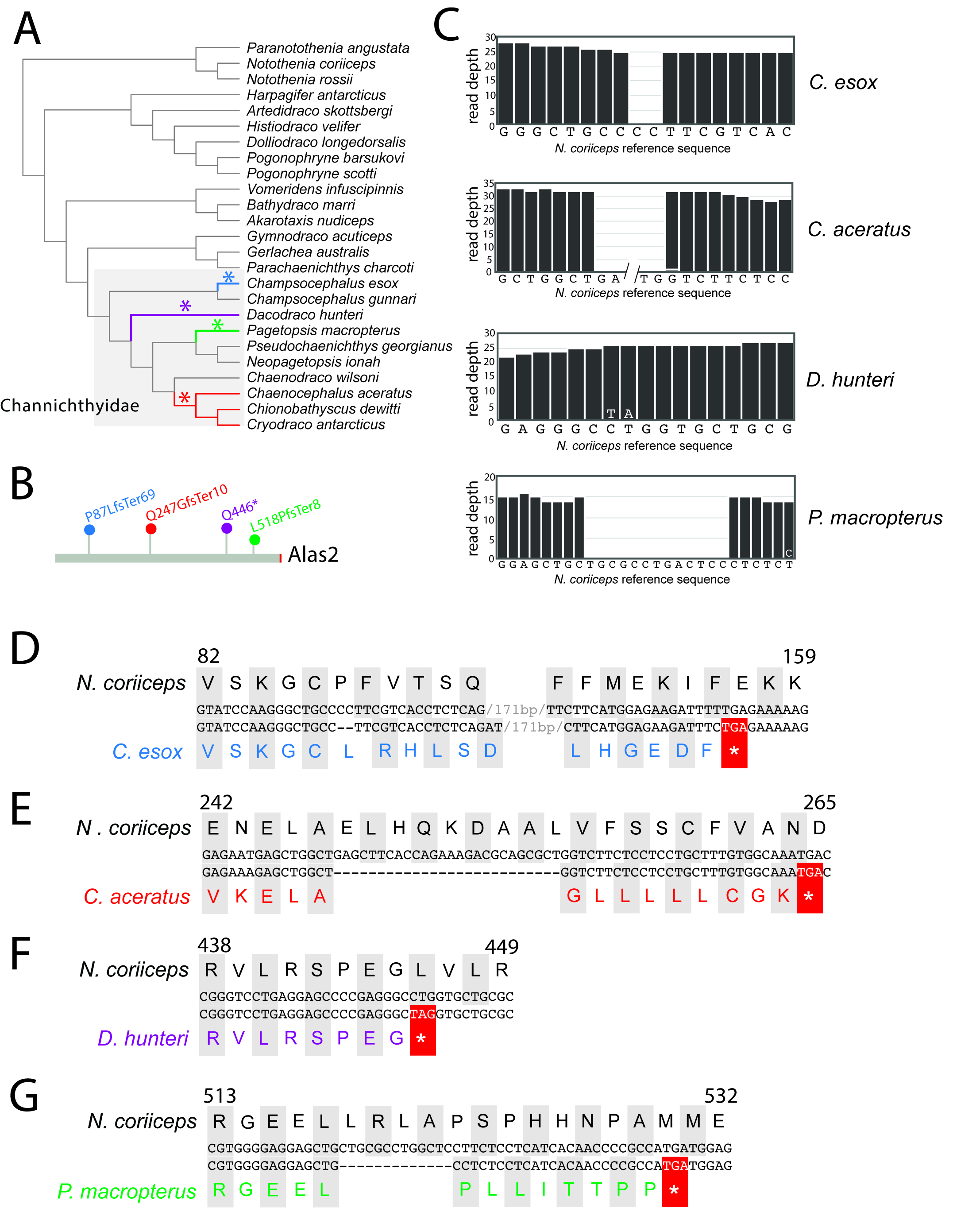

Supplement: S5 Fig — (A) Notothenioid phylogeny showing presence of truncating alleles (*) in four icefish species. (B) Mutant alleles; asterisk color corresponds to branches in A. (C) Sequencing read depth for each species aligned to the Notothenia coriiceps reference genome. Gaps in read depth correspond to deletions in each read relative to the reference genome. (D-G) The icefishes show distinct frameshifts and truncations in Alas2 compared to the N. coriiceps reference sequence. Alignment start/stop coordinates in D-G are based on position in the N. coriiceps genome assembly (XP_010782407.1). (TIF) [file pgen.1009173.s005.tif]

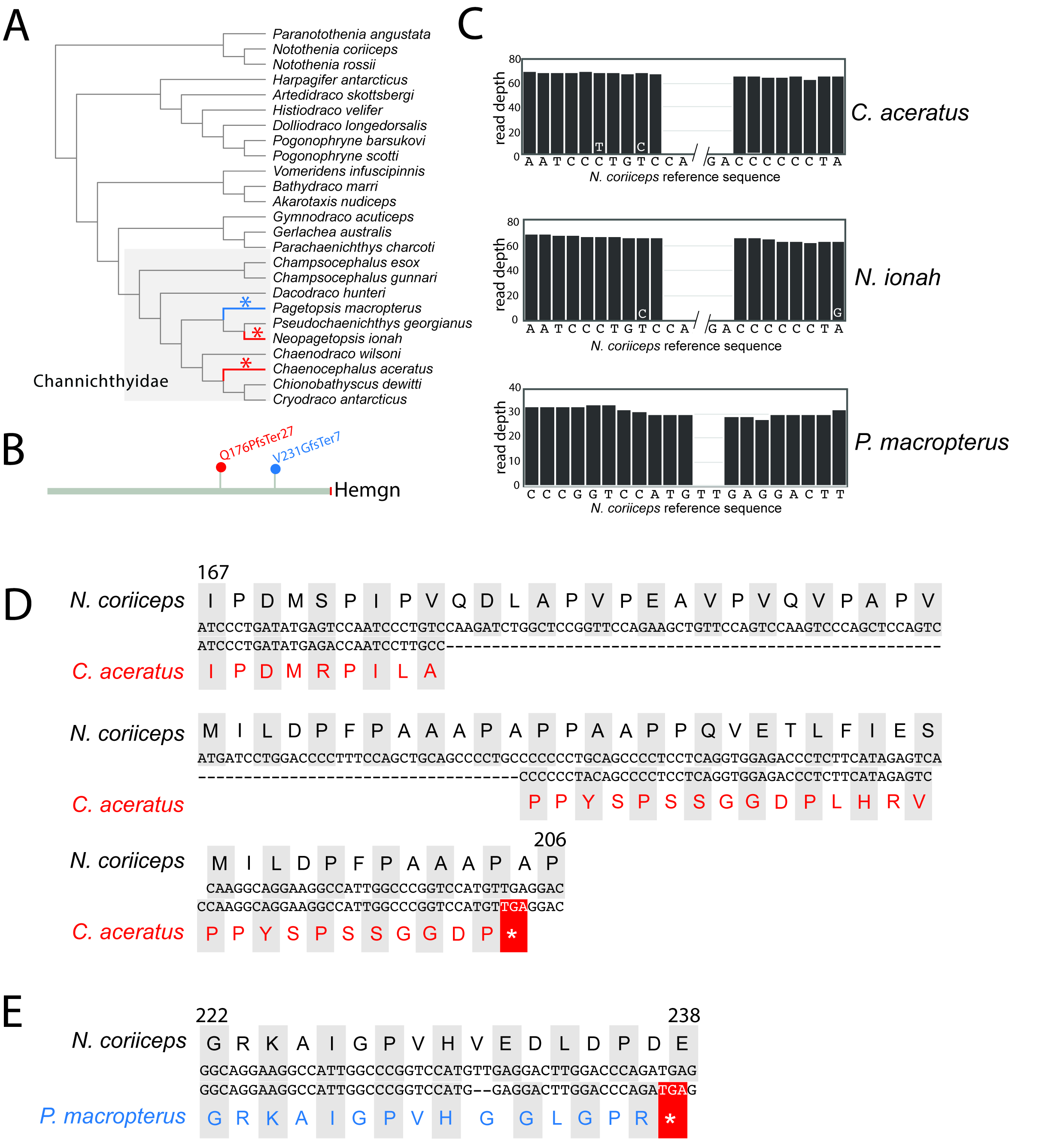

Supplement: S6 Fig — (A) Phylogeny of the notothenioids showing the presence of truncating alleles (*) in three icefish species. (B) Mutant alleles; asterisk color corresponds to branches in A. (C) Sequencing read depth for each species aligned to the Notothenia coriiceps reference genome. Gaps in read depth correspond to deletions in each read relative to the reference genome. (D) Chaenocephalus aceratus and Neopagetopsis ionah show identical frameshifts and truncations in Hemgn compared to the N. coriiceps reference. (E) Pageotopsis macropterus shows a different frameshift and truncation. Alignment start/stop coordinates in D and E are based on position in the N. coriiceps genome assembly (XP_010773828.1). (TIF) [file pgen.1009173.s006.tif]

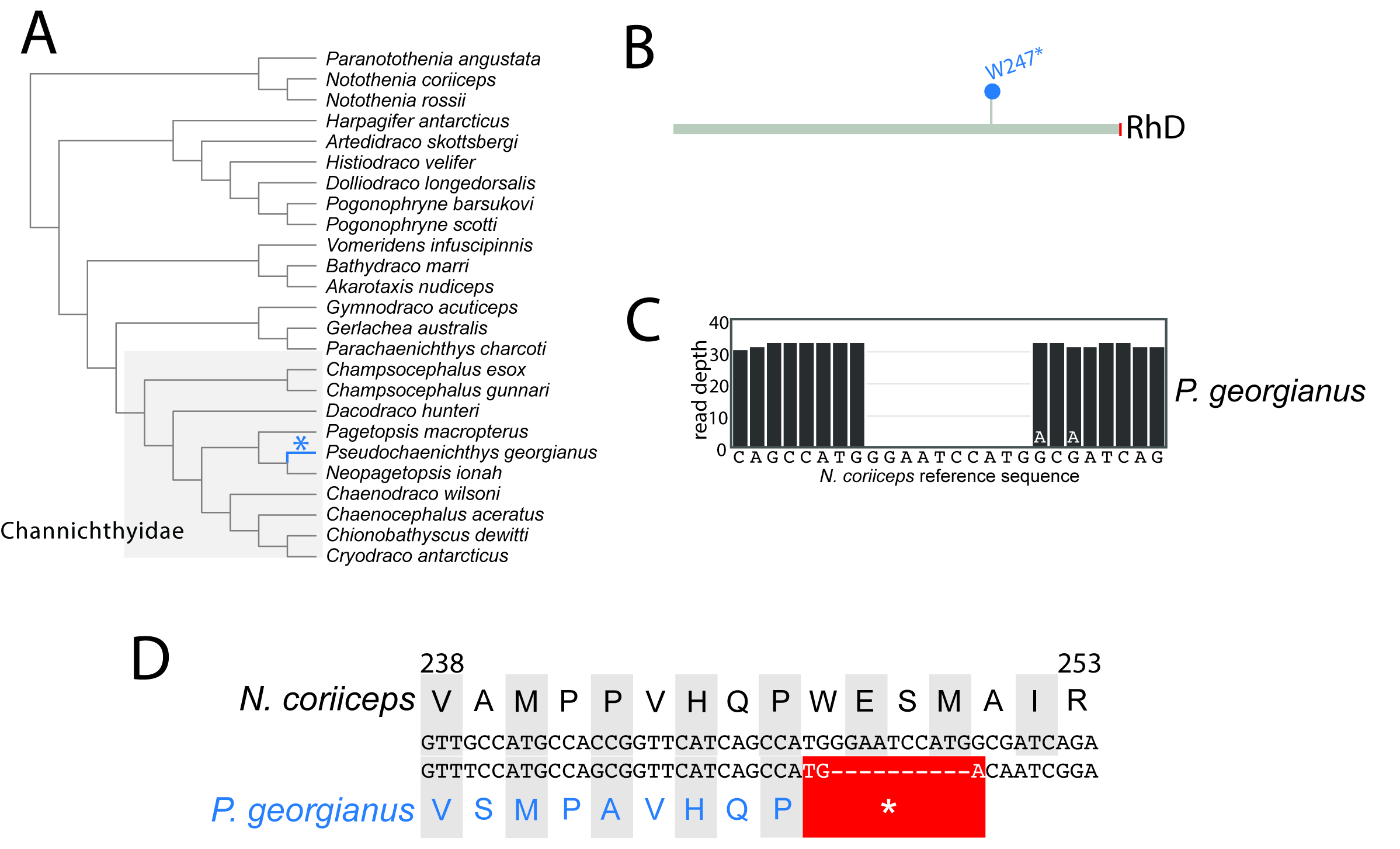

Supplement: S7 Fig — (A) Notothenioid phylogeny showing presence of a truncating allele in P. georgianus (*). (B) The mutation encoded by the allele. (C) Sequencing read depth for P. georgianus as aligned to the Notothenia coriiceps reference genome. The gap in read depth corresponds to a deletion in each read relative to the reference genome. (D) P. georgianus shows a frameshift and truncation in Rhd compared to the N. coriiceps reference sequence. Alignment start/stop coordinates are based on position in the N. coriiceps genome assembly (XP_010782194.1). (TIF) [file pgen.1009173.s007.tif]

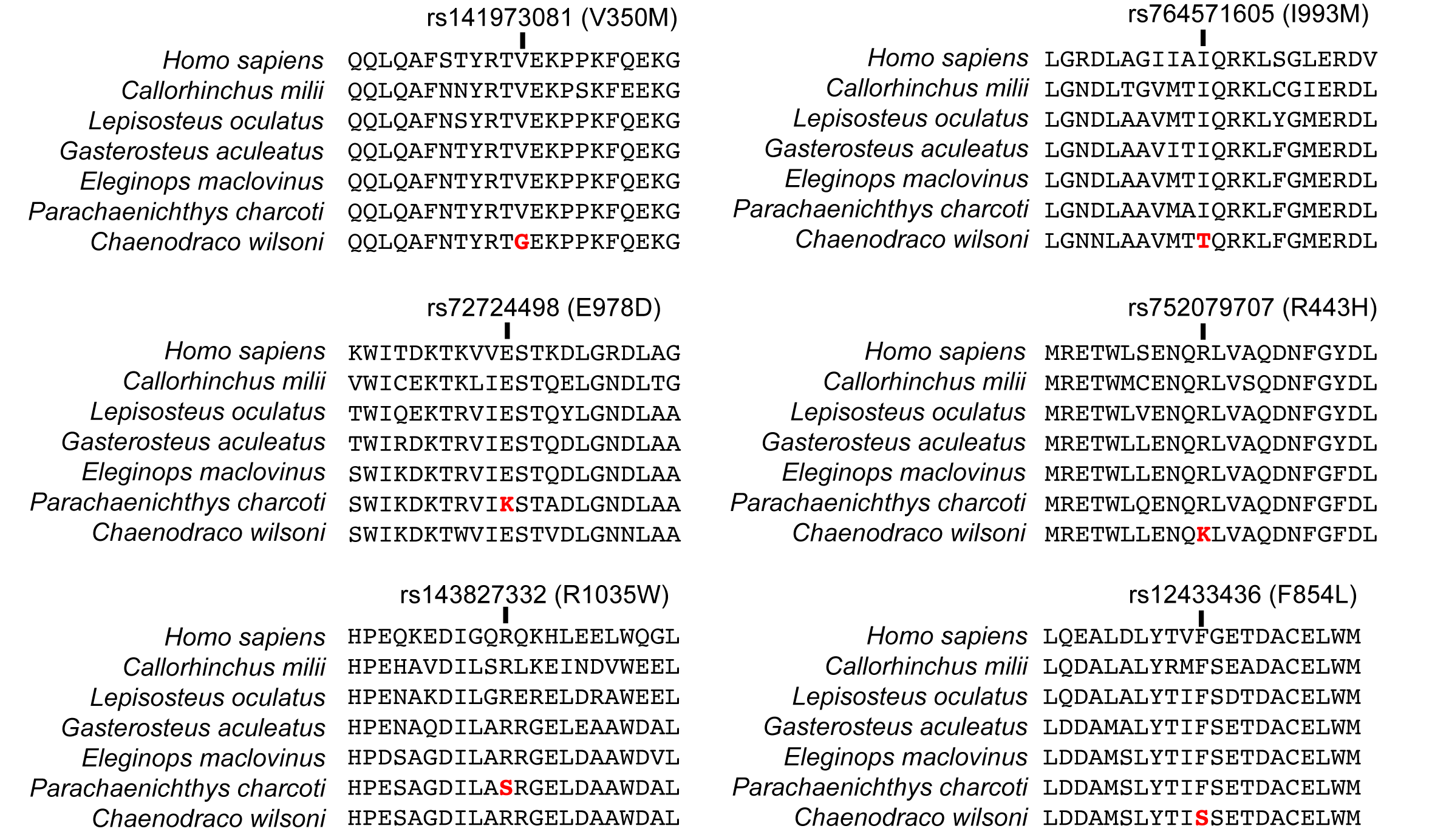

Supplement: S8 Fig — Variant amino acid substitutions in Beta-spectrin of the dragonfish Parachaenichthys charcoti and a representative icefish Chaenodraco wilsoni highlighted in red. Beta-spectrin sequences for three-spined stickleback (Gasterosteus aculeatus), spotted gar (Lepisosteus oculatus), elephant shark (Gallorhinchus milii) and human (Homo sapiens) are provided for comparison. The dbSNP identifier (ClinVar) for deleterious variants found in human patients with spherocytic anemia/elliptocytosis are shown above each alignment. (TIF) [file pgen.1009173.s008.tif]

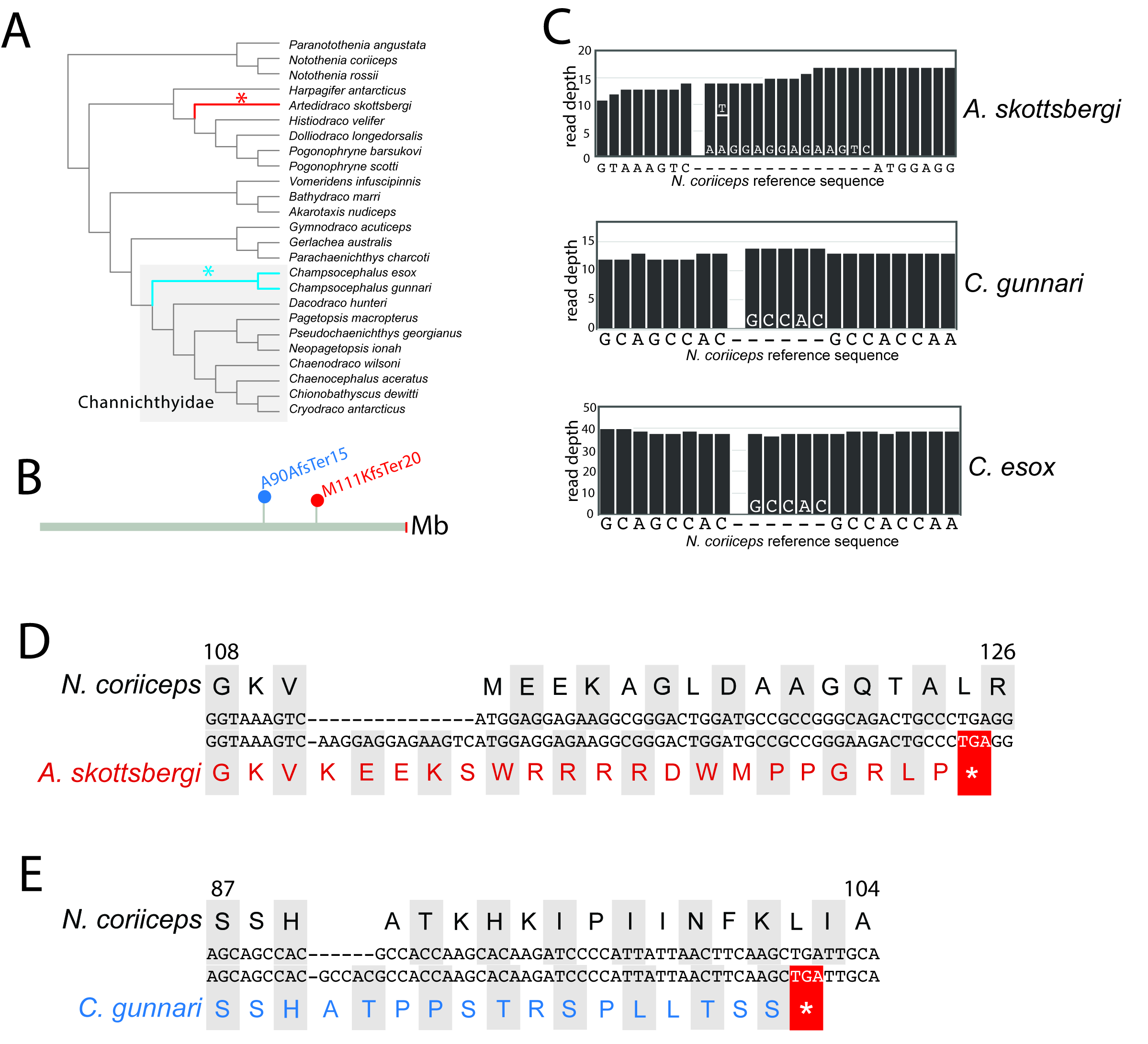

Supplement: S9 Fig — (A) Phylogeny of the notothenioids showing the presence of truncating alleles (*) in three species. (B) Mutant alleles; asterisk color corresponds to branches in A. (C) Sequencing read depth for each species aligned to the Notothenia coriiceps reference genome. Gaps in read depth correspond to deletions in each read relative to the reference genome. (D) Red-blooded species Artedidraco skottsbergi Mb compared to the N. coriiceps reference. (E) Champsocephalus gunnari and C. esox shows identical frameshifts in Mb. Alignment start/stop coordinates in D and E are based on position in the N. coriiceps genome assembly (NP_001290223.1). (TIF) [file pgen.1009173.s009.tif]

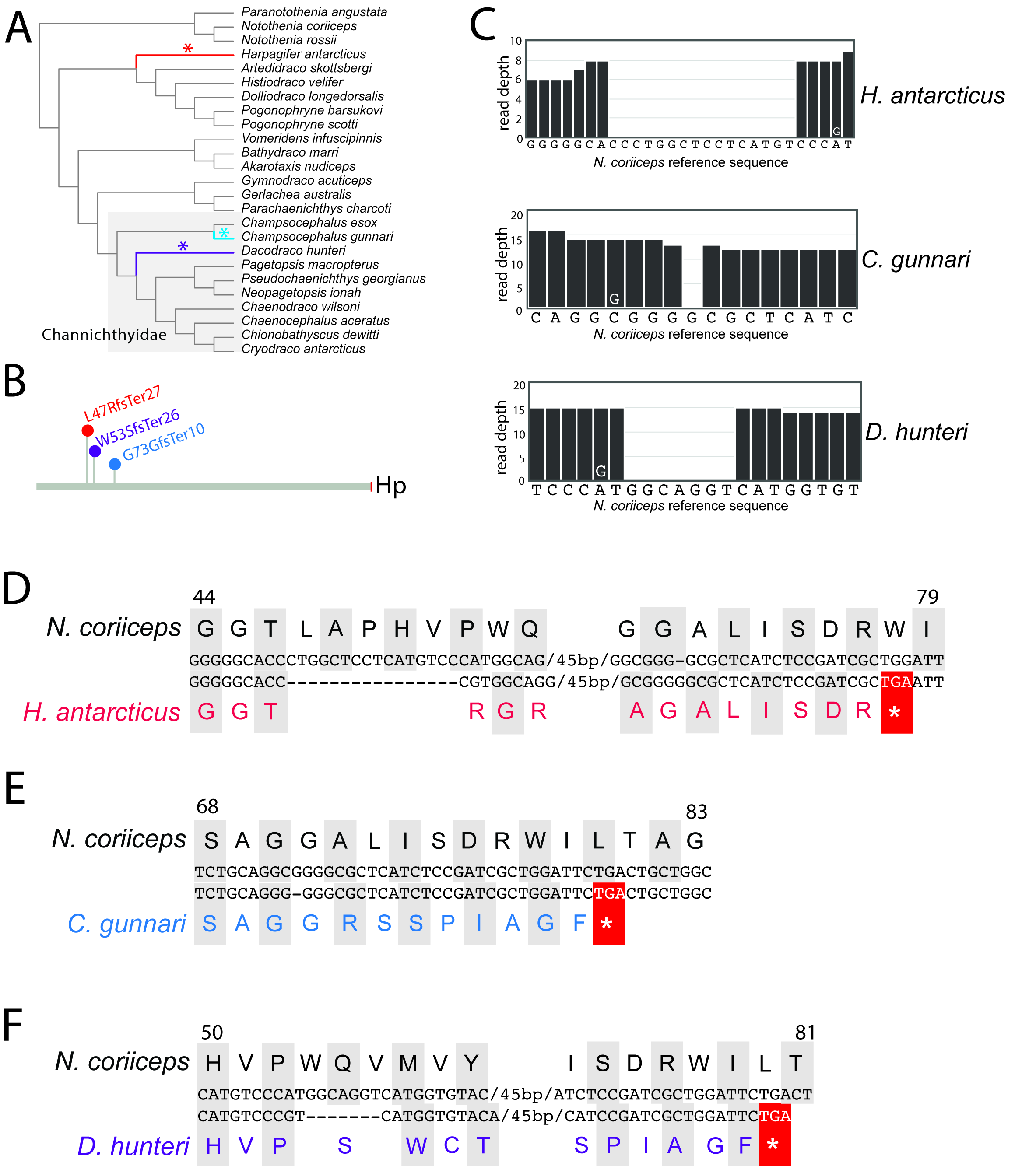

Supplement: S10 Fig — (A) Phylogeny of the notothenioids showing the presence of truncating alleles (*) in three species. (B) Mutant alleles; asterisk color corresponds to branches in A. (C) Sequencing read depth for each species aligned to the Notothenia coriiceps reference genome. Gaps in read depth correspond to deletions in each read relative to the reference genome. (D) Red-blooded species Harpagifer antarcticus Hp compared to the N. coriiceps reference. The icefish species (E) Champsocephalus gunnari and (F) Dacodraco hunteri have different frameshifts and truncations in Hp. Alignment start/stop coordinates in D-F are based on position in the N. coriiceps genome assembly (XP_010770321.1). (TIF) [file pgen.1009173.s010.tif]

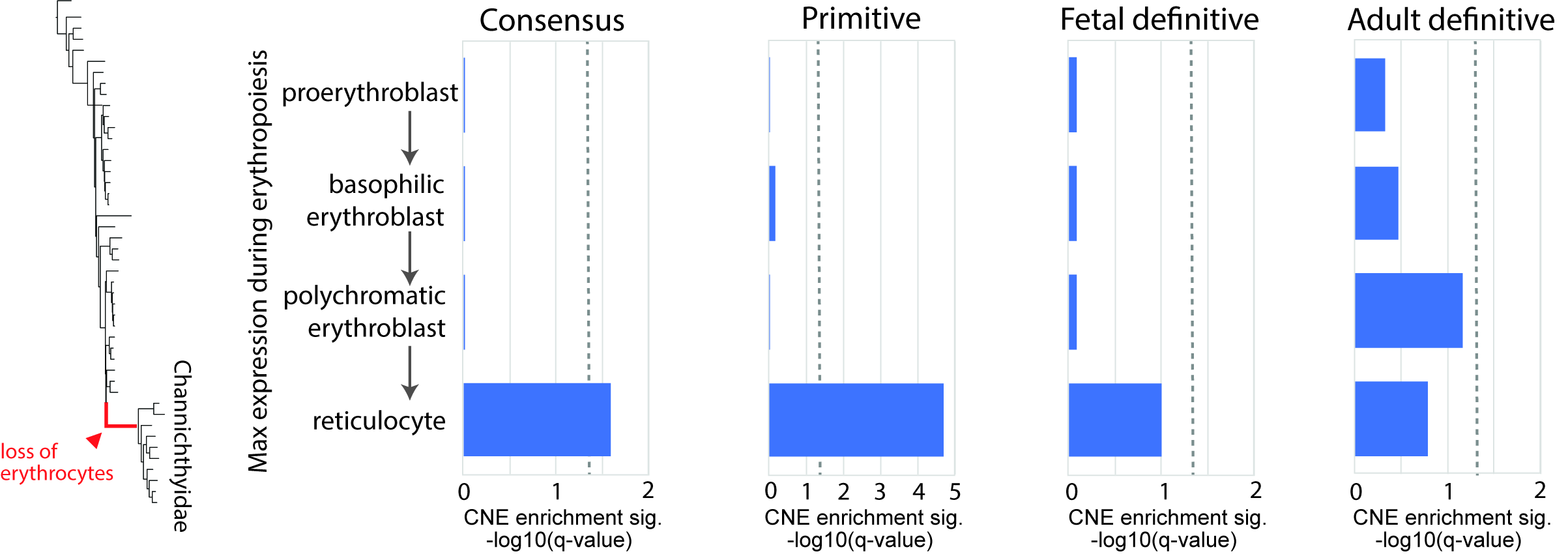

Supplement: S11 Fig — Three waves of mammalian erythropoiesis are defined by distinct patterns of gene expression and (locations): primitive (yolk sac blood island), fetal definitive (liver) and adult definitive (bone marrow). For each erythropoietic wave, accelerated evolution of CNEs near maximally expressed genes is shown for four cellular stages of erythroid differentiation/maturation: proerythroblast, basophilic erythroblast/normoblast, polychromatic erythroblast/normoblast, reticulocyte. The Consensus is the intersection of maximally expressed genes across each the three erythropoietic waves. Dashed line corresponds to q-value of 0.05. Gene expression data from ErythronDB [44]. (TIF) [file pgen.1009173.s011.tif]

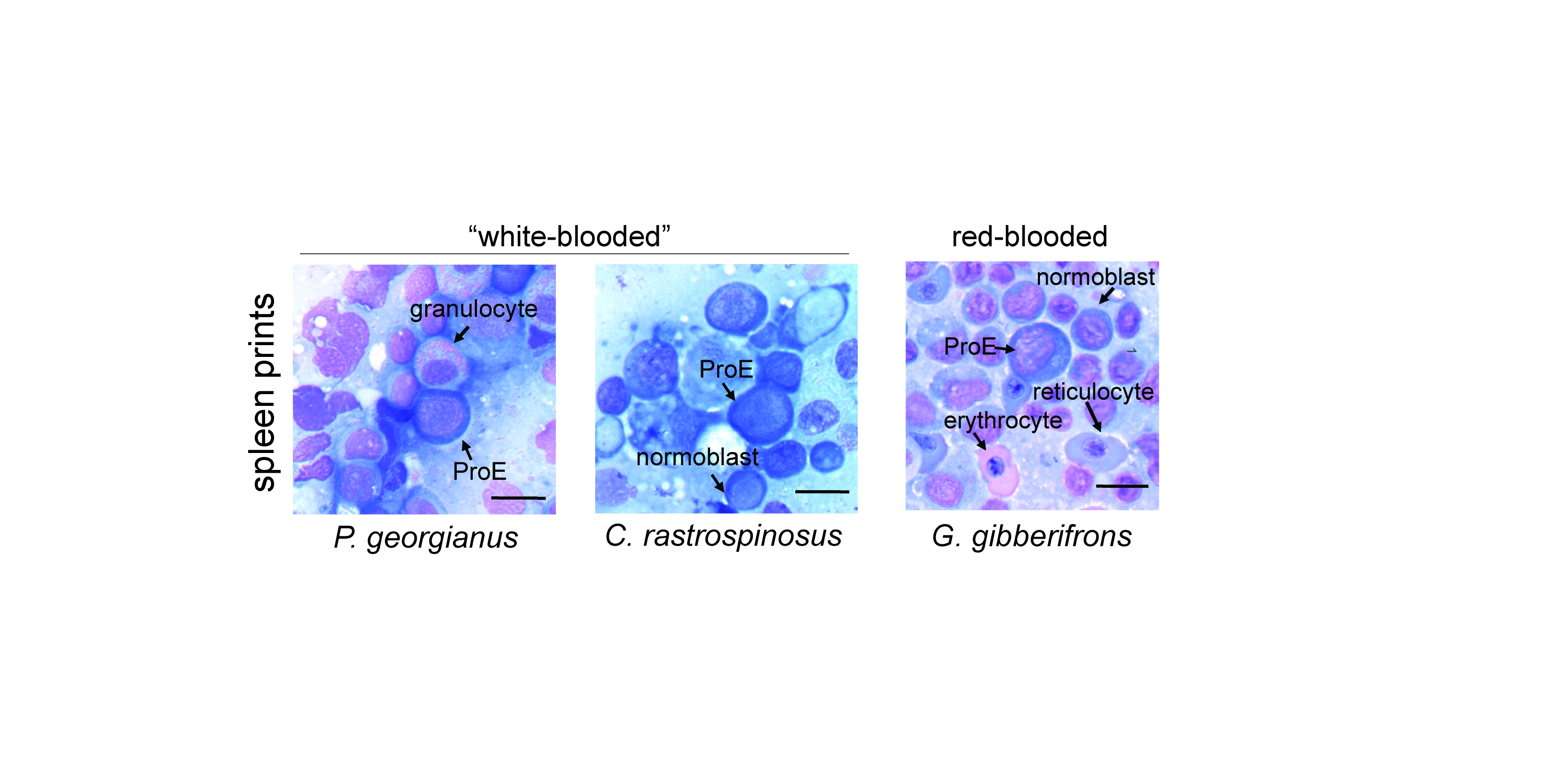

Supplement: S12 Fig — Two “white-blooded” icefishes, Pseudochaenichthys georgianus and Chionodraco rastrospinosus, show the presence of erythroid progenitors [proerythroblasts (ProEs) and normoblasts] but lack later stages of maturation (e.g., reticulocytes, erythrocytes). By contrast, the red-blooded notothen, Gobionotothen gibberifrons, displays the complete erythropoietic progression: ProE → normoblast → reticulocyte → erythrocyte. Scale bar = 10 μm. (TIF) [file pgen.1009173.s012.tif]
