# Supplementary material for: Developmental constraint shaped genome evolution and erythrocyte loss in Antarctic fishes following paleoclimate change
Source: PLoS Genet. 2020 Oct 27;16(10):e1009173. doi: 10.1371/journal.pgen.1009173 (PMC7660546; doi:10.1371/journal.pgen.1009173)
Supplement: S3 Table — (PDF) [file pgen.1009173.s015.pdf]

S3 Table. Relative evolutionary rate and notothenioid biogeography

Distribution†	Species	Family	RER‡
HA	*Pogonophryne barsukovi*	Artedidraconidae	3.160
HA	*Pagetopsis macropterus*	Channichthyidae	2.856
HA	*Histiodraco velifer*	Artedidraconidae	2.783
HA	*Trematomus bernacchii*	Nototheniidae	2.293
HA	*Dacodraco hunteri*	Channichthyidae	1.599
HA	*Vomeridens infuscipinnis*	Bathydraconidae	1.297
HA	*Neopagetopsis ionah*	Channichthyidae	1.163
HA	*Trematomus newnesi*	Nototheniidae	1.155
HA	*Pleuragramma antarctica*	Nototheniidae	1.133
sub-Antarctic	*Cottoperca trigloides*	Bovichtidae	0.927
HA	*Trematomus eulepidotus*	Nototheniidae	0.829
sub-Antarctic	*Paranotothenia angustata*	Nototheniidae	0.692
HA	*Chaenodraco wilsoni*	Channichthyidae	0.460
HA	*Trematomus hansoni*	Nototheniidae	0.344
HA	*Akarotaxis nudiceps*	Nototheniidae	0.224
sub-Antarctic	*Bovichtus diacanthus*	Bovichtidae	0.073
HA	*Cryodraco antarcticus*	Channichthyidae	-0.002
sub-Antarctic	*Champsocephalus esox*	Channichthyidae	-0.167
HA	*Parachaenichthys charcoti*	Bathydraconidae	-0.195
HA	*Pogonophryne scotti*	Artedidraconidae	-0.234
HA	*Aethotaxis mitopteryx*	Nototheniidae	-0.280
HA	*Trematomus borchgrevinki*	Nototheniidae	-0.339
HA	*Notothenia coriiceps*	Nototheniidae	-0.477
HA	*Gerlachea australis*	Bathydraconidae	-0.487
HA	*Trematomus scotti*	Nototheniidae	-0.499
HA	*Dissostichus mawsoni*	Nototheniidae	-0.612
HA	*Lepidonotothen squamifrons*	Nototheniidae	-0.617
sub-Antarctic	*Patagonotothen guntheri*	Nototheniidae	-0.810
sub-Antarctic	*Patagonotothen cornucola*	Nototheniidae	-0.942
HA	*Bathydraco marri*	Bathydraconidae	-1.044
sub-Antarctic	*Dissostichus eleginoides*	Nototheniidae	-1.078
HA	*Chionobathyscus dewitti*	Channichthyidae	-1.152

Distribution†	Species	Family	RER‡
HA	*Gymnodraco acuticeps*	Bathydraconidae	-1.210
sub-Antarctic	*Eleginops maclovinus*	Eleginopsidae	-1.639
HA	*Dolloidraco longedorsalis*	Artedidraconidae	-1.893
HA	*Artedidraco skottsbergi*	Artedidraconidae	-1.943
sub-Antarctic	*Pseudaphritis urvillii*	Pseudaphritidae	-2.063

† HA - high latitude Antarctic

‡ Relative evolutionary rate across all conserved non-coding elements flanking human anemia-associated genes (HP:0001903)
