# Supplementary material for: Developmental constraint shaped genome evolution and erythrocyte loss in Antarctic fishes following paleoclimate change
Source: PLoS Genet. 2020 Oct 27;16(10):e1009173. doi: 10.1371/journal.pgen.1009173 (PMC7660546; doi:10.1371/journal.pgen.1009173)
Supplement: S4 Table — (PDF) [file pgen.1009173.s016.pdf]

S4 Table. Coverage and mutations in candidate erythrocyte genes

Ensembl ID	Gene Name	Type	Avg Coverage†	Avg Coverage Icefish†	FE marrow	FE erythrocytes	Pleiotropy Score	Truncating variant (s) ‡
ENSGACG00000006807	Alas2	heme and hemoglobin biosynthesis	97.8%	100.0%	6.58	2.09	0	✓
ENSGACG00000001091	Dmtn	cytoskeleton	91.2%	94.1%	0.37	6.30	0	
ENSGACG00000017373	Hemgn	development/transcription factors	95.7%	100.0%	1.61	5.18	0	✓
ENSGACG00000013918	Hbb	heme and hemoglobin biosynthesis	78.9%	13.0%	8.59	2.70	0	✓
ENSGACG00000014492	Hba	heme and hemoglobin biosynthesis	81.0%	37.2%	4.99	2.56	0	✓
ENSGACG00000009865	Rhag	membrane proteins and solute transporters	71.9%	73.7%	23.77	6.82	0	
ENSGACG00000015628	Gypc	cytoskeleton	81.6%	100.0%	1.37	1.72	0	
ENSGACG00000007369	Rhd	membrane proteins and solute transporters	94.3%	98.4%	5.54	8.88	0	✓
ENSGACG00000007574	Slc25a28	membrane proteins and solute transporters	96.8%	99.8%	0.58	0.32	0	
ENSGACG00000001549	Tfcp2	development/transcription factors	96.1%	99.5%	0.16	0.69	0	
ENSGACG00000009512	Eif2ak1	heme and hemoglobin biosynthesis	73.6%	74.5%	0.49	1.69	1	
ENSGACG00000007530	Slc25a38	membrane proteins and solute transporters	96.1%	100.0%	0.54	3.16	1	
ENSGACG00000007468	Car6	carbonic anhydrases	97.8%	100.0%	0.00	0.25	1	
ENSGACG00000007890	Car3	carbonic anhydrases	74.8%	80.9%	0.00	0.18	1	
ENSGACG00000016482	Hmgb2	development/transcription factors	95.5%	100.0%	3.16	0.78	1	
ENSGACG00000002257	Kdm1b	development/transcription factors	97.8%	100.0%	0.40	0.13	1	
ENSGACG00000008971	Isg15	development/transcription factors	62.2%	63.1%	0.67	0.57	1	
ENSGACG00000012154	Aqp9	membrane proteins and solute transporters	97.5%	100.0%	0.11	0.57	2	
ENSGACG00000010765	Slc25a37	membrane proteins and solute transporters	91.2%	93.4%	1.64	0.94	2	
ENSGACG00000014377	Urod	heme and hemoglobin biosynthesis	97.7%	100.0%	0.91	3.53	2	
ENSGACG00000003213	Car5b	carbonic anhydrases	95.5%	99.9%	0.07	0.03	2	
ENSGACG00000015396	Car7	carbonic anhydrases	97.3%	100.0%	0.00	1.00	2	
ENSGACG00000018021	Alad	heme and hemoglobin biosynthesis	97.7%	100.0%	0.33	5.84	3	
ENSGACG00000001433	Epb41	cytoskeleton	95.0%	98.2%	0.80	0.56	3	
ENSGACG00000001495	Trf	heme and hemoglobin biosynthesis	96.2%	99.7%	0.00	0.01	3	
ENSGACG00000004180	Ppox	heme and hemoglobin biosynthesis	83.2%	87.7%	0.44	2.33	3	
ENSGACG00000018597	Tmod1	cytoskeleton	91.5%	94.5%	0.15	1.25	3	
ENSGACG00000012574	Slc2a1	membrane proteins and solute transporters	96.0%	99.9%	0.17	0.02	3	
ENSGACG00000011803	Car14	carbonic anhydrases	96.9%	99.9%	0.02	0.03	3	
ENSGACG00000018705	Chd4	development/transcription factors	96.5%	99.5%	0.33	0.35	3	
ENSGACG00000020462	Crebrf	development/transcription factors	96.8%	100.0%	1.12	0.14	4	
ENSGACG00000018134	Gfi1b	development/transcription factors	97.1%	100.0%	15.87	2.75	4	
ENSGACG00000009442	Nfe2	development/transcription factors	97.2%	100.0%	5.76	0.27	4	
ENSGACG00000013189	Add2	cytoskeleton	90.4%	91.2%	0.90	7.34	4	
ENSGACG00000008037	Ftl1	heme and hemoglobin biosynthesis	97.5%	100.0%	0.34	0.32	4	
ENSGACG00000003384	Xk	membrane proteins and solute transporters	97.8%	99.9%	0.70	2.18	4	
ENSGACG00000019222	Bcl11a	development/transcription factors	97.5%	100.0%	0.26	0.65	4	

Ensembl ID	Gene Name	Type	Avg Coverage†	Avg Coverage Icefish†	FE marrow	FE erythrocytes	Pleiotropy Score	Truncating variant (s) ‡
ENSGACG00000019143	Klf1	development/transcription factors	96.0%	100.0%	25.70	7.32	5	
ENSGACG00000009874	Lmo2	development/transcription factors	97.7%	100.0%	0.66	0.49	5	
ENSGACG00000016373	Tfrc	heme and hemoglobin biosynthesis	94.8%	100.0%	1.10	1.98	6	
ENSGACG00000010082	Cpox	heme and hemoglobin biosynthesis	97.6%	100.0%	1.04	6.33	6	
ENSGACG00000011054	Myb	development/transcription factors	96.3%	99.9%	0.77	0.20	6	
ENSGACG00000002554	Zfpm1	development/transcription factors	96.5%	98.7%	0.42	0.81	6	
ENSGACG00000005173	Hmbs	heme and hemoglobin biosynthesis	94.7%	97.3%	5.39	5.59	6	
ENSGACG00000012004	Flvcr1	heme and hemoglobin biosynthesis	55.7%	63.1%	0.19	0.58	6	
ENSGACG00000013741	Dntm1	development/transcription factors	97.2%	99.8%	0.54	0.50	6	
ENSGACG00000006591	Ets1	development/transcription factors	97.5%	100.0%	0.07	0.00	6	
ENSGACG00000013704	Jak2	development/transcription factors	97.5%	100.0%	0.51	0.43	6	
ENSGACG00000017365	Jak2	development/transcription factors	97.5%	100.0%	0.51	0.43	6	
ENSGACG00000009622	Slc4a1	membrane proteins and solute transporters	92.1%	94.8%	7.93	2.74	7	
ENSGACG00000015484	Fth1	heme and hemoglobin biosynthesis	96.5%	100.0%	1.18	0.60	7	
ENSGACG00000000651	Fech	heme and hemoglobin biosynthesis	94.1%	96.8%	2.25	4.07	8	
ENSGACG00000018336	Ldb1	development/transcription factors	96.8%	99.8%	0.44	0.60	8	
ENSGACG00000015635	Sox6	development/transcription factors	97.8%	100.0%	0.64	1.11	8	
ENSGACG00000010218	Gata1	development/transcription factors	96.7%	100.0%	19.53	2.31	8	
ENSGACG00000002608	Uros	heme and hemoglobin biosynthesis	95.4%	100.0%	0.51	5.32	8	
ENSGACG00000004999	Car2	carbonic anhydrases	96.9%	99.0%	0.14	4.73	8	
ENSGACG00000009013	Acvr1ba	development/transcription factors	97.7%	100.0%	0.15	0.01	8	
ENSGACG00000000719	Acvr1bb	development/transcription factors	97.8%	100.0%	0.15	0.01	8	
ENSGACG00000017383	Aqp1	membrane proteins and solute transporters	97.8%	100.0%	0.07	5.00	9	
ENSGACG00000009608	Gata2	development/transcription factors	96.9%	99.7%	0.04	0.05	9	
ENSGACG00000013846	Tal1	development/transcription factors	97.6%	100.0%	1.77	1.03	9	
ENSGACG00000011100	Sptb	cytoskeleton	95.1%	97.5%	0.89	5.58	10	
ENSGACG00000009679	Kdm1a	development/transcription factors	97.7%	100.0%	0.21	0.42	10	
ENSGACG00000008634	Stat5a	development/transcription factors	97.6%	100.0%	0.49	0.46	10	
ENSGACG00000015405	Stat5b	development/transcription factors	97.1%	99.8%	0.38	0.19	11	
ENSGACG00000009373	Kitl	development/transcription factors	95.7%	100.0%	0.01	0.01	12	
ENSGACG00000017699	Ank1	cytoskeleton	97.1%	100.0%	0.14	6.67	12	
ENSGACG00000015083	Acvr2a	development/transcription factors	97.7%	99.6%	0.09	0.11	13	
ENSGACG00000015589	Stat1	development/transcription factors	96.2%	99.6%	0.19	0.14	13	
ENSGACG00000008910	Foxo3	development/transcription factors	98.1%	100.0%	0.81	0.47	16	

† Average coverage across dataset at a minimum depth of 4x reads

‡ Whole gene deletion or truncating variant (nonsense, frameshift) in at least one icefish lineage
