# Supplementary material for: Developmental constraint shaped genome evolution and erythrocyte loss in Antarctic fishes following paleoclimate change
Source: PLoS Genet. 2020 Oct 27;16(10):e1009173. doi: 10.1371/journal.pgen.1009173 (PMC7660546; doi:10.1371/journal.pgen.1009173)
Supplement: S5 Table — (PDF) [file pgen.1009173.s017.pdf]

S5 Table. Coverage and mutations in erythroid-biased genes

Ensembl ID	Gene Name	Avg Coverage†	Avg Coverage Icefish†	FE marrow	FE erythrocytes	Pleiotropy Score	Truncating variant (s) ‡
ENSGACG00000000027	Mcm2	93.3%	96.4%	1.27	1.18	11	
ENSGACG00000004199	Hdgf	97.9%	100.0%	1.06	1.46	1	
ENSGACG00000006776	Mcm5	72.1%	69.9%	1.05	1.41	1	
ENSGACG00000009622	Slc4a1	92.1%	94.8%	7.93	2.74	7	
ENSGACG00000007018	Slc4a1	96.7%	98.4%	7.93	2.74	7	
ENSGACG00000002407	Cdt1	94.1%	98.8%	4.34	1.45	0	
ENSGACG00000003179	Timm23	97.8%	100.0%	1.34	1.02	4	
ENSGACG00000018996	Usp15	97.5%	99.9%	1.81	1.83	5	
ENSGACG00000004862	Josd1	83.2%	86.6%	1.04	1.62	1	
ENSGACG00000010082	Cpox	97.6%	100.0%	1.04	6.33	6	
ENSGACG00000016373	Tfrc	94.8%	100.0%	1.10	1.98	6	
ENSGACG00000005398	Tfrc	95.4%	100.0%	1.10	1.98	6	
ENSGACG00000009865	Rhag	71.9%	73.7%	23.77	6.82	0	
ENSGACG00000000651	Fech	94.1%	96.8%	2.25	4.07	8	
ENSGACG00000006807	Alas2	97.8%	100.0%	6.58	2.09	0	✓
ENSGACG00000020793	Rabgef1	68.1%	71.0%	1.14	2.15	4	
ENSGACG00000013350	Pecam1	95.5%	99.9%	6.11	5.67	0	
ENSGACG00000019062	Tk1	97.0%	100.0%	1.19	1.70	10	
ENSGACG00000014938	Pigq	96.9%	99.8%	1.37	3.27	1	
ENSGACG00000019155	Mcm10	96.3%	100.0%	1.02	1.33	2	
ENSGACG00000018134	Gfi1b	97.1%	100.0%	15.87	2.75	4	
ENSGACG00000017832	Clp1	97.9%	100.0%	1.17	1.21	8	
ENSGACG00000005726	Pcna	97.8%	100.0%	1.20	1.06	5	
ENSGACG00000017373	Hemgn	95.7%	100.0%	1.61	5.18	0	✓
ENSGACG00000005437	Orc1	81.5%	86.2%	2.27	2.43	1	
ENSGACG00000013846	Tal1	97.6%	100.0%	1.77	1.03	9	
ENSGACG00000007369	Rhd	94.3%	98.4%	5.54	8.88	0	✓
ENSGACG00000010218	Gata1	96.7%	100.0%	19.53	2.31	8	
ENSGACG00000016176	Abcb10	89.8%	92.4%	1.97	3.94	3	
ENSGACG00000005173	Hmbs	94.7%	97.3%	5.39	5.59	6	
ENSGACG00000012552	Blvrb	97.1%	100.0%	2.17	2.85	2	
ENSGACG00000004430	Rpia	97.3%	100.0%	1.14	1.63	2	
ENSGACG00000019143	Klf1	96.0%	100.0%	25.70	7.32	5	
ENSGACG00000014492	Hba-a1	79.9%	37.2%	4.99	2.56	0	✓
ENSGACG00000004078	Fastkd5	97.4%	100.0%	1.20	1.27	4	
ENSGACG00000015628	Gypc	81.6%	100.0%	1.37	1.72	0	
ENSGACG00000013918	Hbb	79.9%	13.6%	8.59	2.70	0	✓

† Average coverage across dataset at a minimum depth of 4x reads

‡ Whole gene deletion or truncating variant (nonsense, frameshift) in at least one icefish lineage
