# Supplementary material for: Developmental constraint shaped genome evolution and erythrocyte loss in Antarctic fishes following paleoclimate change
Source: PLoS Genet. 2020 Oct 27;16(10):e1009173. doi: 10.1371/journal.pgen.1009173 (PMC7660546; doi:10.1371/journal.pgen.1009173)
Supplement: S6 Table — (PDF) [file pgen.1009173.s018.pdf]

S6 Table. Excluded terms from Mammalian Phenotype Ontology (MP) in pleiotropy analysis

MP Term	Name
MP:0000202	abnormal circulating alkaline phosphatase level
MP:0000208	decreased hematocrit
MP:0000215	absent erythrocytes
MP:0000226	abnormal mean corpuscular volume
MP:0000233	abnormal blood flow velocity
MP:0000237	obsolete decreased blood cell number
MP:0000245	abnormal erythropoiesis
MP:0000248	macrocytosis
MP:0000256	echinocytosis
MP:0000314	schistocytosis
MP:0000315	hemoglobinuria
MP:0000332	hemoglobinemia
MP:0000348	abnormal aerobic fitness
MP:0000603	pale liver
MP:0000689	abnormal spleen morphology
MP:0000734	muscle hypoplasia
MP:0000748	progressive muscle weakness
MP:0000752	dystrophic muscle
MP:0000759	abnormal skeletal muscle morphology
MP:0001189	absent skin pigmentation
MP:0001190	reddish skin
MP:0001191	abnormal skin condition
MP:0001201	translucent skin
MP:0001264	increased body size
MP:0001265	decreased body size
MP:0001569	abnormal circulating bilirubin level
MP:0001574	abnormal oxygen level
MP:0001577	anemia
MP:0001585	hemolytic anemia
MP:0001586	abnormal erythrocyte cell number
MP:0001588	abnormal hemoglobin
MP:0001589	abnormal mean corpuscular hemoglobin
MP:0001598	abnormal blood viscosity
MP:0001599	abnormal blood volume
MP:0001697	abnormal embryo size
MP:0001698	decreased embryo size

MP:0001699	increased embryo size
MP:0001721	absent visceral yolk sac blood islands
MP:0001722	pale yolk sac
MP:0001730	embryonic growth arrest
MP:0001731	abnormal postnatal growth
MP:0001732	postnatal growth retardation
MP:0001770	abnormal iron level
MP:0001786	skin edema
MP:0001933	abnormal litter size
MP:0001934	increased litter size
MP:0001935	decreased litter size
MP:0002088	abnormal embryonic growth/weight/body size
MP:0002089	abnormal postnatal growth/weight/body size
MP:0002095	abnormal skin pigmentation
MP:0002106	abnormal muscle physiology
MP:0002108	abnormal muscle morphology
MP:0002224	abnormal spleen size
MP:0002225	obsolete abnormal spleen cellularity
MP:0002227	abnormal spleen capsule morphology
MP:0002228	abnormal spleen trabecular vein morphology
MP:0002288	obsolete litter size
MP:0002319	hyperoxia
MP:0002329	abnormal blood gas level
MP:0002354	abnormal spleen trabecular artery morphology
MP:0002355	obsolete abnormal spleen venous sinus
MP:0002356	abnormal spleen red pulp morphology
MP:0002357	abnormal spleen white pulp morphology
MP:0002358	abnormal spleen periarteriolar lymphoid sheath morphology
MP:0002359	abnormal spleen germinal center morphology
MP:0002361	abnormal spleen central arteriole morphology
MP:0002362	abnormal spleen marginal zone morphology
MP:0002363	abnormal spleen marginal sinus morphology
MP:0002424	abnormal reticulocyte morphology
MP:0002447	abnormal erythrocyte morphology
MP:0002591	decreased mean corpuscular volume
MP:0002592	obsolete mean erythrocyte count traits
MP:0002593	high mean erythrocyte cell number
MP:0002594	low mean erythrocyte cell number
MP:0002596	abnormal hematocrit
MP:0002608	increased hematocrit

MP:0002640	reticulocytosis
MP:0002641	anisopoikilocytosis
MP:0002642	anisocytosis
MP:0002643	poikilocytosis
MP:0002810	microcytic anemia
MP:0002811	macrocytic anemia
MP:0002812	spherocytosis
MP:0002813	microcytosis
MP:0002814	hyperchromasia
MP:0002874	decreased hemoglobin content
MP:0002875	decreased erythrocyte cell number
MP:0002897	blotchy skin
MP:0002954	obsolete abnormal aerobic energy metabolism
MP:0002966	decreased circulating alkaline phosphatase level
MP:0002968	increased circulating alkaline phosphatase level
MP:0003015	abnormal circulating bicarbonate level
MP:0003016	increased circulating bicarbonate level
MP:0003017	decreased circulating bicarbonate level
MP:0003060	increased aerobic running capacity
MP:0003131	increased erythrocyte cell number
MP:0003342	accessory spleen
MP:0003396	abnormal embryonic hematopoiesis
MP:0003656	abnormal erythrocyte physiology
MP:0003657	abnormal erythrocyte osmotic lysis
MP:0003717	pallor
MP:0003852	skeletal muscle necrosis
MP:0003956	abnormal body size
MP:0003984	embryonic growth retardation
MP:0004142	abnormal muscle tone
MP:0004143	muscle hypertonia
MP:0004151	decreased circulating iron level
MP:0004152	abnormal circulating iron level
MP:0004196	abnormal prenatal growth/weight/body size
MP:0004197	abnormal fetal growth/weight/body size
MP:0004198	abnormal fetal size
MP:0004199	increased fetal size
MP:0004200	decreased fetal size
MP:0004201	fetal growth retardation
MP:0004229	abnormal embryonic erythropoiesis
MP:0004230	abnormal embryonic erythrocyte morphology

MP:0004232	decreased muscle weight
MP:0004233	abnormal muscle weight
MP:0004797	increased anti-erythrocyte antigen antibody level
MP:0004817	abnormal skeletal muscle mass
MP:0004818	increased skeletal muscle mass
MP:0004819	decreased skeletal muscle mass
MP:0004827	increased susceptibility to autoimmune hemolytic anemia
MP:0004828	decreased susceptibility to autoimmune hemolytic anemia
MP:0004846	absent skeletal muscle
MP:0004951	abnormal spleen weight
MP:0004952	increased spleen weight
MP:0004953	decreased spleen weight
MP:0004969	pale kidney
MP:0005028	abnormal trophectoderm morphology
MP:0005097	polychromatophilia
MP:0005152	pancytopenia
MP:0005288	abnormal oxygen consumption
MP:0005289	increased oxygen consumption
MP:0005290	decreased oxygen consumption
MP:0005344	increased circulating bilirubin level
MP:0005369	muscle phenotype
MP:0005406	abnormal heart size
MP:0005505	thrombocytosis
MP:0005561	increased mean corpuscular hemoglobin
MP:0005562	decreased mean corpuscular hemoglobin
MP:0005563	abnormal hemoglobin content
MP:0005564	increased hemoglobin content
MP:0005635	decreased circulating bilirubin level
MP:0005637	abnormal iron homeostasis
MP:0005640	abnormal mean corpuscular hemoglobin concentration
MP:0005641	increased mean corpuscular hemoglobin concentration
MP:0005642	decreased mean corpuscular hemoglobin concentration
MP:0005649	increased spleen neoplasm incidence
MP:0006034	myoglobinuria
MP:0006208	lethality throughout fetal growth and development
MP:0006351	abnormal glycosylated hemoglobin level
MP:0006352	decreased glycosylated hemoglobin level
MP:0006353	increased glycosylated hemoglobin level
MP:0008234	absent spleen marginal zone
MP:0008387	hypochromic anemia

MP:0008388	hypochromic microcytic anemia
MP:0008389	hypochromic macrocytic anemia
MP:0008473	abnormal spleen follicular dendritic cell network
MP:0008474	absent spleen germinal center
MP:0008475	intermingled spleen red and white pulp
MP:0008476	increased spleen red pulp amount
MP:0008477	decreased spleen red pulp amount
MP:0008478	increased spleen white pulp amount
MP:0008479	decreased spleen white pulp amount
MP:0008481	increased spleen germinal center number
MP:0008482	decreased spleen germinal center number
MP:0008483	increased spleen germinal center size
MP:0008484	decreased spleen germinal center size
MP:0008737	abnormal spleen physiology
MP:0008738	abnormal liver iron level
MP:0008739	abnormal spleen iron level
MP:0008740	abnormal intestinal iron level
MP:0008741	abnormal heart iron level
MP:0008742	abnormal kidney iron level
MP:0008743	decreased liver iron level
MP:0008772	increased heart ventricle size
MP:0008807	increased liver iron level
MP:0008808	decreased spleen iron level
MP:0008809	increased spleen iron level
MP:0008810	increased circulating iron level
MP:0008849	abnormal hemoglobin concentration distribution width
MP:0008850	increased hemoglobin concentration distribution width
MP:0008851	decreased hemoglobin concentration distribution width
MP:0008941	reticulocytopenia
MP:0008945	hyperchromic macrocytic anemia
MP:0008954	abnormal cellular hemoglobin content
MP:0008955	increased cellular hemoglobin content
MP:0008956	decreased cellular hemoglobin content
MP:0008962	abnormal carbon dioxide production
MP:0008963	increased carbon dioxide production
MP:0008964	decreased carbon dioxide production
MP:0009246	pale spleen
MP:0009323	abnormal spleen development
MP:0009395	increased nucleated erythrocyte cell number
MP:0009398	abnormal skeletal muscle fiber size

MP:0009399	increased skeletal muscle fiber size
MP:0009403	increased variability of skeletal muscle fiber size
MP:0009405	increased skeletal muscle fiber number
MP:0009406	decreased skeletal muscle fiber number
MP:0009408	decreased skeletal muscle fiber density
MP:0009409	abnormal skeletal muscle fiber type ratio
MP:0009410	abnormal skeletal muscle satellite cell proliferation
MP:0009411	abnormal skeletal muscle fiber triad morphology
MP:0009412	skeletal muscle fiber degeneration
MP:0009413	skeletal muscle fiber atrophy
MP:0009414	skeletal muscle fiber necrosis
MP:0009415	skeletal muscle degeneration
MP:0009416	cardiac muscle degeneration
MP:0009417	skeletal muscle atrophy
MP:0009418	cardiac muscle atrophy
MP:0009458	abnormal skeletal muscle size
MP:0009459	skeletal muscle hyperplasia
MP:0009460	skeletal muscle hypoplasia
MP:0009461	skeletal muscle hypertrophy
MP:0009462	skeletal muscle hypotrophy
MP:0009547	elliptocytosis
MP:0009568	abnormal red blood cell deformability
MP:0009642	abnormal blood homeostasis
MP:0009701	abnormal birth body size
MP:0009702	increased birth body size
MP:0009703	decreased birth body size
MP:0009841	foam cell reticulosis
MP:0009931	abnormal skin appearance
MP:0010020	spleen vascular congestion
MP:0010034	abnormal erythrocyte clearance
MP:0010035	increased erythrocyte clearance
MP:0010036	decreased erythrocyte clearance
MP:0010067	increased red blood cell distribution width
MP:0010068	decreased red blood cell distribution width
MP:0010074	stomatocytosis
MP:0010175	leptocytosis
MP:0010176	dacryocytosis
MP:0010177	acanthocytosis
MP:0010178	increased number of Howell-Jolly bodies
MP:0010237	abnormal skeletal muscle weight

MP:0010238	increased skeletal muscle weight
MP:0010239	decreased skeletal muscle weight
MP:0010240	decreased skeletal muscle size
MP:0010245	abnormal spleen perifollicular zone morphology
MP:0010375	increased kidney iron level
MP:0010376	decreased kidney iron level
MP:0010399	decreased skeletal muscle glycogen level
MP:0010401	increased skeletal muscle glycogen level
MP:0010563	increased heart right ventricle size
MP:0010577	abnormal heart right ventricle size
MP:0010579	increased heart left ventricle size
MP:0010580	decreased heart left ventricle size
MP:0010630	abnormal cardiac muscle tissue morphology
MP:0010632	cardiac muscle necrosis
MP:0010696	increased siderocyte number
MP:0010832	lethality during fetal growth through weaning
MP:0010865	prenatal growth retardation
MP:0010866	abnormal prenatal body size
MP:0010957	abnormal aerobic respiration
MP:0011089	perinatal lethality, complete penetrance
MP:0011091	prenatal lethality
MP:0011098	embryonic lethality during organogenesis, complete penetrance
MP:0011099	lethality throughout fetal growth and development, complete penetrance
MP:0011101	prenatal lethality, incomplete penetrance
MP:0011109	lethality throughout fetal growth and development, incomplete penetrance
MP:0011111	lethality during fetal growth through weaning, complete penetrance
MP:0011112	lethality during fetal growth through weaning, incomplete penetrance
MP:0011171	increased number of Heinz bodies
MP:0011188	increased erythrocyte protoporphyrin level
MP:0011204	abnormal visceral yolk sac blood island morphology
MP:0011235	abnormal blood oxygen capacity
MP:0011236	increased blood oxygen capacity
MP:0011237	decreased blood oxygen capacity
MP:0011239	abnormal skin coloration
MP:0011240	abnormal fetal derived definitive erythrocyte morphology
MP:0011241	abnormal fetal derived definitive erythrocyte cell number
MP:0011242	increased fetal derived definitive erythrocyte cell number
MP:0011243	decreased fetal derived definitive erythrocyte cell number

MP:0011244	absent fetal derived definitive erythrocytes
MP:0011245	abnormal fetal derived definitive erythrocyte physiology
MP:0011263	abnormal spleen mesenchyme morphology
MP:0011514	skin hemorrhage
MP:0011519	abnormal placenta labyrinth size
MP:0011520	increased placental labyrinth size
MP:0011521	decreased placental labyrinth size
MP:0011526	abnormal placenta fetal blood space morphology
MP:0011630	increased mitochondria size
MP:0011631	decreased mitochondria size
MP:0011890	increased circulating ferritin level
MP:0011891	decreased circulating ferritin level
MP:0011892	abnormal circulating transferrin level
MP:0011893	increased circulating transferrin level
MP:0011894	decreased circulating transferrin level
MP:0011895	abnormal circulating unsaturated transferrin level
MP:0011896	increased circulating unsaturated transferrin level
MP:0011897	decreased circulating unsaturated transferrin level
MP:0011913	abnormal reticulocyte cell number
MP:0011992	increased erythrocyte catalase activity
MP:0012056	abnormal polar trophectoderm morphology
MP:0012057	abnormal mural trophectoderm morphology
MP:0012102	absent trophectoderm
MP:0012115	abnormal trophectoderm cell proliferation
MP:0012116	increased trophectoderm cell proliferation
MP:0012117	decreased trophectoderm cell proliferation
MP:0012118	absent trophectoderm cell proliferation
MP:0012119	increased trophectoderm apoptosis
MP:0012120	trophectoderm cell degeneration
MP:0012363	abnormal erythrocyte sodium level
MP:0012364	decreased erythrocyte sodium level
MP:0012365	increased erythrocyte sodium level
MP:0012366	abnormal erythrocyte magnesium level
MP:0012367	decreased erythrocyte magnesium level
MP:0012368	increased erythrocyte magnesium level
MP:0012369	abnormal erythrocyte potassium level
MP:0012370	decreased erythrocyte potassium level
MP:0012371	increased erythrocyte potassium level
MP:0012372	abnormal erythrocyte ion content
MP:0012373	abnormal erythrocyte magnesium ion content

MP:0012374	decreased erythrocyte magnesium ion content
MP:0012375	increased erythrocyte magnesium ion content
MP:0012376	abnormal erythrocyte potassium ion content
MP:0012377	decreased erythrocyte potassium ion content
MP:0012378	increased erythrocyte potassium ion content
MP:0012379	abnormal erythrocyte sodium ion content
MP:0012380	decreased erythrocyte sodium ion content
MP:0012381	increased erythrocyte sodium ion content
MP:0012384	abnormal erythrocyte ion transport
MP:0012385	abnormal erythrocyte potassium:chloride symporter activity
MP:0012386	decreased erythrocyte potassium:chloride symporter activity
MP:0012387	increased erythrocyte potassium:chloride symporter activity
MP:0012388	abnormal erythrocyte sodium:hydrogen antiporter activity
MP:0012389	decreased erythrocyte sodium:hydrogen antiporter activity
MP:0012390	increased erythrocyte sodium:hydrogen antiporter activity
MP:0012391	abnormal erythrocyte sodium:potassium-exchanging ATPase activity
MP:0012392	decreased erythrocyte sodium:potassium-exchanging ATPase activity
MP:0012393	increased erythrocyte sodium:potassium-exchanging ATPase activity
MP:0012394	abnormal erythrocyte calcium-activated potassium channel activity
MP:0012395	decreased erythrocyte calcium-activated potassium channel activity
MP:0012396	increased erythrocyte calcium-activated potassium channel activity
MP:0012397	abnormal nucleated erythrocyte cell number
MP:0012398	decreased nucleated erythrocyte cell number
MP:0012650	abnormal erythrocyte catalase level
MP:0012653	decreased erythrocyte catalase level
MP:0012656	increased erythrocyte catalase level
MP:0012663	decreased haptoglobin level
MP:0012664	decreased circulating haptoglobin level
MP:0012665	increased haptoglobin level
MP:0012666	increased circulating haptoglobin level
MP:0013215	abnormal haptoglobin level
MP:0013301	abnormal pancreas iron level
MP:0013302	increased pancreas iron level
MP:0013303	decreased pancreas iron level
MP:0013403	abnormal circulating lactate level
MP:0013404	decreased circulating lactate level
MP:0013405	increased circulating lactate level
MP:0013657	abnormal blood cell morphology
MP:0020240	increased skeletal muscle cell apoptosis
MP:0020241	decreased skeletal muscle cell apoptosis

MP:0020323	abnormal heart apex size
MP:0020365	increased brain iron level
MP:0020366	decreased brain iron level
MP:0020367	increased heart iron level
MP:0020368	decreased heart iron level
MP:0020369	increased intestinal iron level
MP:0020453	abnormal erythrocyte aggregation
MP:0020454	decreased erythrocyte aggregation
MP:0020455	increased erythrocyte aggregation
MP:0020825	ectopic spleen
